# Nutrients and Bioactive Compounds from *Cannabis sativa* Seeds: A Review Focused on Omics-Based Investigations

**DOI:** 10.3390/ijms26115219

**Published:** 2025-05-29

**Authors:** Tiziana M. Sirangelo, Gianfranco Diretto, Alessia Fiore, Simona Felletti, Tatiana Chenet, Martina Catani, Natasha Damiana Spadafora

**Affiliations:** 1 Division Biotechnologies, ENEA—Italian National Agency for New Technologies, Energy and Sustainable Economic Development, Casaccia Research Center, Via Anguillarese 301, 00123 Rome, Italy; gianfranco.diretto@enea.it (G.D.); alessia.fiore@enea.it (A.F.); 2 Department of Environmental and Prevention Sciences, University of Ferrara, Via L. Borsari 46, 44121 Ferrara, Italy; simona.felletti@unife.it (S.F.); tatiana.chenet@unife.it (T.C.); 3 Department of Chemical, Pharmaceutical and Agricultural Sciences, University of Ferrara, Via L. Borsari 46, 44121 Ferrara, Italy; martina.catani@unife.it

**Keywords:** *Cannabis sativa*, hemp, hemp seeds, nutrients, bioactive compounds, omics, multi-omics

## Abstract

Hemp (*Cannabis sativa* L.) is a versatile crop that can be processed to obtain different products with multiple applications. Its seeds are a well-documented ancient source of proteins, fibers and fats, all of which possess high nutritional value. Additionally, metabolites such as flavones and phenols are present in the seeds, contributing to their antioxidant properties. Due to hemp seeds’ distinctive nutritional profile, the interest in exploring the potential use in food and nutraceuticals is growing, and they can be considered an interesting and promising alternative resource for human and animal feeding. Omics studies on hemp seeds and their by-products are also being developed, and they contribute to improving our knowledge about the genome, transcriptome, proteome, metabolome/lipidome, and ionome of these sustainable food resources. This review illustrates the main nutrients and bioactive compounds of hemp seeds and explores the most relevant omics techniques and investigations related to them. It also addresses the various products derived from processing the whole seed, such as oil, dehulled seeds, hulls, flour, cakes, meals, and proteins. Moreover, this work discusses research aimed at elucidating the molecular mechanisms underlying their protein, lipid, fiber, and metabolic profile. The advantages of using omics and multi-omics approaches to highlight the nutritional values of hemp seed by-products are also discussed. In our opinion, this work represents an excellent starting point for researchers interested in studying hemp seeds as source of nutrients and bioactive compounds from a multi-level molecular perspective.

## 1. Introduction

Hemp (*Cannabis sativa* L.) is the oldest cultivated crop belonging to the Cannabaceae family, considered to be a multipurpose crop thanks to its morphological variability and versatility [1,2]. In fact, its seeds are used for nutrients, medicines and fuels, stalks for tissues, and inflorescences for medicines [1]. Hemp contains several cannabinoids, including Δ^9^-tetrahydrocannabinol (THC) and cannabidiol (CBD), which are the most investigated secondary metabolites produced by cannabis among the ~250 identified ones [3,4]. According to the THC content, hemp can be categorized into two types, the drug type (THC > 0.3%) and non-drug type (THC < 0.3%) [4]. This crop has been widely used as a fiber source for a long time, but the non-drug type or industrial hemp can be used for food, feed, and also for therapeutic purposes [5].

**Figure 1:**
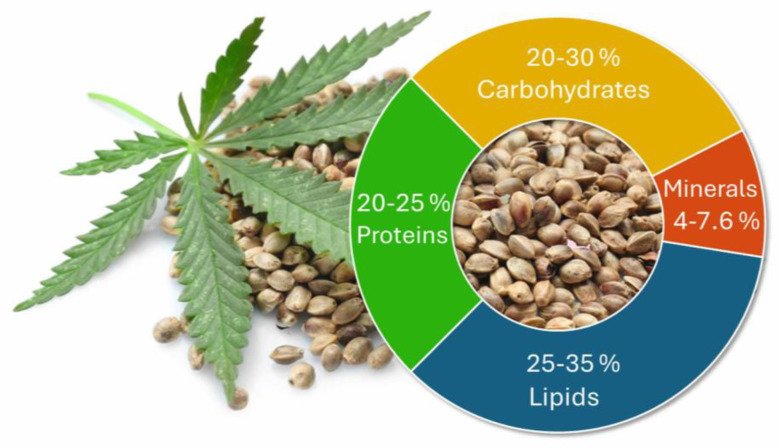
The most relevant nutrients in hemp seeds.

Hemp seeds, which are commonly referred to as seeds from industrial hemp, are a well-documented natural source of proteins, carbohydrates, and fats with a high nutritional value (Figure 1) [6,7]. Whole hemp seeds contain 20–25% proteins [8,9], 20–30% carbohydrates [5,6,10], whose values are comparable to those found in whole flax (*Linum usitatissimum* L.) seeds [11], and 25–35% fats [10,12]. They also contain fat-soluble vitamins, such as vitamin A and E [6,7], and minerals, like potassium, magnesium, and zinc (4–7.6%) [7,13,14]. A substantial amount of polyunsaturated fatty acids (PUFAs), that are not commonly found in vegetable oils, has also been reported. These include linoleic (ω-6) and α-linolenic acids (ω-3), comprising approximately 50% and 20% of the total fatty acids, respectively [6,15].

Metabolites, such as flavones and phenols, are also contained in the seeds, contributing to their antioxidant properties [16,17], with beneficial effects on human health, particularly on the cardiovascular system and/or the immune response, as well as preventing diabetes [18,19,20].

In recent years, research into the potential use of industrial hemp seeds in food and nutraceuticals has increased due to their distinctive nutritional profile, confirming their role as a promising alternative and sustainable resource for human and animal nutrition [21]. For instance, hemp seeds can be processed as a whole [16], reduced to flour [22], or transformed into oil [23,24]. Oil is the main component of hemp seed processing, with pressed cake and meal considered by-products. However, these by-products are now used to enrich foods to obtain functional foods, due to their chemical and nutritional values [25]. Indeed, they contain high amounts of bioactive compounds, carbohydrates, lipids, organic acids, proteins, vitamins, and minerals, that can be isolated and added in foods as well as in pharmaceutics [11,26,27].

Nevertheless, today, residual material from hemp seeds processing is often considered waste and used as biomass [28]. This overlooks the potential value of this residual material, which could be transformed into resources for new products. Adopting this approach allows for the sustainable use of hemp seeds and their by-products, meeting the current needs to reduce food waste and implement circular economy strategies [29,30,31,32].

Omics studies on hemp have been growing in recent years. The recent easing legislation regulating its cultivation has promoted cannabis research, along with the availability of its genome sequences, which has become a focus of the scientific community [33,34,35,36]. In addition, gene expression investigations have also been crucial for a better understanding of the cannabinoid metabolic pathways [37,38]; similarly, several metabolomics [12,39,40] and proteomics investigations have been carried out on hemp seeds [41,42,43] and their products [44,45,46], contributing to improving our knowledge about the proteome and the metabolome of these sustainable food resources. Multi-omics studies have also demonstrated that this interdisciplinary approach is a powerful tool to detect correlations between biological processes and metabolic pathways across different omics layers, and also to explore novel metabolites with therapeutic potential [36,47].

This work illustrates the main nutrients and bioactive components of hemp seeds, as well as the by-products derived from them. Omics and multi-omics techniques and studies aimed to investigate hemp seed nutritional and bioactive profile are also reported and discussed.

The review was conducted following a narrative approach to provide a comprehensive overview of the existing literature. The selection of references was based on a combination of strategies to ensure relevance and accuracy. Specifically, we prioritized articles published in recent refereed journals dating back to about 15 years ago, that highlight omics and multi-omics research on the nutritional properties of hemp seeds and their by-products. To identify these articles, we conducted a search using relevant keywords such as “hemp seed”, “*Cannabis sativa*”, “nutrients”, “bioactive compounds”, “omics”, and “multi-omics” across databases, including PubMed, Scopus, and Web of Science. In addition to recent publications, we also included key publications and highly cited articles that are considered foundational in the field, ensuring that the review provides historical context and acknowledges key research milestones.

This work represents in our opinion a potential resource for future molecular biology research focused on this natural source of nutrients and bioactive compounds.

## 2. Hemp Seed Nutrients and Bioactive Compounds

Numerous studies have investigated the chemical composition of hemp seeds, highlighting a rich profile of fatty acids, proteins, oils, and phenolics [7,24,25,48,49,50,51]. Despite extensive research, the complexity of these components has so far precluded a complete and exhaustive characterization. Notably, the content of these constituents can vary significantly among different cultivars [39,52], different environments, and agronomic conditions [17,53].

Particularly, the year of cultivation (growing season) significantly influences (*p* ≤ 0.05) the contents of lipids, total dietary fiber, and fatty acids, such as γ-linolenic, linoleic, α-linolenic, oleic, and stearic acid [54]; conversely, protein content is not affected by growing season [53]. In contrast, the profiles of proteins, dietary fiber, lipids, and fatty acids strongly varied between growing year and genotype [53]. The impact of environmental conditions on the metabolite content of hemp seeds was also highlighted [17].

Overall, plant genotype and environmental conditions can affect the quantity and quality of macronutrients and phytochemicals in hemp seeds, thereby influencing their nutraceutical properties [42].

Table 1 summarizes the concentration, biological effects, and references for the most abundant oils, polysaccharides, proteins, vitamins, and minerals in hemp seeds.

### 2.1. Proteins

Whole hemp seeds typically contain approximately 25% proteins, and this percentage increases in dehulled seeds due to the removal of dietary fiber-rich outer shells [55]. This protein content is higher than or comparable to other protein-rich seeds, such as quinoa seeds (~13%) and flax seeds (~21%) [9,56], making hemp seeds a valuable addition to food products.

Furthermore, hemp seed proteins are easily digestible and contain an adequate amount of all the essential amino acids [25,57], providing sufficient amounts of all 9 essential amino acids, including those required for the nutritional needs of two-to-five-year-old children [58]. The most abundant amino acid is the glutamic acid, followed by arginine [59], a non-essential amino acid that plays a crucial role in the production of nitric oxide contributing to vasodilatation and improving human blood circulation [60,61]. In addition, hemp seed proteins provide high quantities of sulfur-containing amino acids, such as methionine and cystine, which may contribute to enhanced antioxidant properties [9,62].

The hemp seed protein content mainly includes two types of storage proteins [63] with exceptional nutritional potential: globulins (predominantly edestin) and albumins [64,65]. Edestin, a salt-soluble globulin, constitutes a substantial portion of the total storage protein in hemp seeds, ranging from 60% to 75% [9,14]. This protein, classified within the legume family, is rich in essential amino acids [6,66]. Hemp seeds edestins have 11S globulin features and, on the basis of their amino acid composition, are generally categorized into three groups (type1, type2, and type3) [64,67]. All three protein types were very rich in arginine and glutamic acid, with edestin type2 particularly rich in methionine residues and type3 edestin also abundant in cysteine and methionine [64,67], suggesting that these proteins can be used to improve the nutritional quality of plant-based food.

On the other hand, albumin (2S albumin), a water-soluble protein, comprises the second major fraction of hemp seeds storage proteins, accounting for approximately 25–30% [14]. Meanwhile, 7S vicilin-like protein is the least abundant storage protein, typically around 5% [9].

Therefore, hemp seed protein products are suitable for inclusion in diets designed for individuals with low-protein intakes and lactose intolerance, as well as for vegans and vegetarians. Moreover, these proteins have been increasingly incorporated into food products such as bakery items, milk alternatives, and meat substitutes [68,69,70,71].

The enzymatic hydrolysis of hemp seed proteins using enzymes such as alcalase, pepsin, or trypsin produces bioactive peptides. These proteins derived have demonstrated potent antioxidant activity, a subject of significant research interest in recent years. Aiello et al. [72] reported on the hydrolysis of hemp seed proteins by pancreatin, pepsin, trypsin, and a mixture of these enzymes. Pancreatin achieved the highest degree of hydrolysis (DH) at 47.5%, followed by trypsin (46.6%), the enzyme mixture (34%), and pepsin (19.7%). The study also revealed a direct correlation between peptide yields and DH values, with each hydrolysate exhibiting a distinct protein and peptide composition.

Taking into account these findings, hemp seed protein hydrolysates show promise as versatile ingredients in functional foods. However, the bioavailability of hemp seed peptides remains an area requiring further investigation, a challenge also observed with peptides from other plant sources [73].

### 2.2. Lipids

Hemp seeds oils contain up to 90% unsaturated fatty acids [39], of which 70% to over 80% is composed by PUFAs [10], and are characterized by an optimal ω-6/ω-3 ratio of 3:1 between, according to the European Food Safety Agency (EFSA) recommendations [74]. Linoleic acid (LA) and α-linolenic acid (ALA) are precursors to ω-6 and ω-3 PUFAs and are known as essential fatty acids (EFAs), since they cannot be synthesized by mammals and must be included in the diet, and are also able to prevent several diseases [10,75,76].

The long-chain PUFAs arachidonic acid (AA), derived from LA conversion, and docosahexaenoic acid (DHA) and eicosapentaenoic acid (EPA), derived from ALA, are the biologically active forms in humans. These PUFAs are essential for various physiological processes, such as maintaining cell membrane structure, supporting cardiovascular health, and regulating metabolic and inflammatory processes through the synthesis of prostaglandin and leukotriene, maintaining skin integrity, and ensuring proper brain function. Several studies [39,53] have reported that the Finola cultivar exhibits the highest ALA content. In addition to LA and ALA, hemp seed oil contains stearidonic acid (SDA), a precursor of other fatty acids, and the γ-linolenic acid (GLA), both of which show anti-inflammatory properties. Specifically, SDA can increase the level of EPA in erythrocytes and plasma phospholipids, contributing to the anti-inflammatory effects [10]. GLA is rapidly converted to Dihomo-γ-Linolenic Acid (DGLA), which is located in the cell membrane, where it serves as a precursor for anti-inflammatory metabolites [77]. Notably, the Finola cultivar contains high amounts of SDA and GLA [10].

Furthermore, the unsaponifiable fraction of hemp seed oil contains tocopherols, including the isomers α-, β-, γ-, and δ-tocopherol. These are the primary antioxidants in hemp seed oil protecting it from oxidation by scavenging free radicals [10,78].

Overall, the unique fatty acid profile and antioxidant content of hemp seed oil make it a valuable dietary component for promoting overall health and well-being.

### 2.3. Carbohydrates

The whole hemp seed contains approximately 28% polysaccharides, of which about 5.5% are water-soluble and 22% are insoluble polysaccharides, while the defatted hemp seed meal contains approximately 42.5% total polysaccharides [11].

Despite the substantial amount of polysaccharides in hemp seed, the nature and the content of the non-cellulosic polysaccharides, including xylan, xyloglucan, and pectin, remain relatively understudied. These polysaccharides contribute to dietary fiber, defined as the part of plant material resistant to enzymatic digestion, which is important for human health.

Dietary fiber has been shown to improve insulin sensitivity, reduce appetite and food intake (thus decreasing the risk of obesity and diabetes), and lower total blood cholesterol. Furthermore, gut microbiota fermentation of dietary fiber produces short-chain fatty acids with anti-carcinogenic and anti-inflammatory properties [79]. In hemp seeds, several studies have reported that dietary fiber constitutes a significant portion of total carbohydrates, with the insoluble fraction being predominant [6,56]. Therefore, hemp seed dietary fibers deserve attention as a valuable ingredient to enhance the fiber content of foods products. The inclusion of hemp seed polysaccharides in the diet can thus offer multiple benefits, particularly in promoting gut health and metabolic regulation.

### 2.4. Vitamins and Minerals

Lipids determine the type of fat-soluble vitamins present in hemp seeds [14,80]. The most abundant vitamins are vitamin E (tocopherols), which helps mitigate oxidative stress, and vitamin A (mostly β-carotene), essential for healthy skin development [14,81]. In addition, the B vitamin group, which contributes to the maintenance of a healthy nervous system, is also well-represented in hemp seeds [7].

Among vitamin E vitamers, γ-tocopherol is the most abundant, followed by α- and δ-tocopherols [82,83]. These compounds are known to preserve the oxidative stability of hemp seed oils and positively affect their storage [84].

Several recent studies have quantified tocopherols in different hemp seed oils. Izzo et al. [85] reported tocopherol content ranging between 3.47 and 13.25 mg/100 g across cultivars from different geographic regions. Similar findings were related by Aiello et al. [86] and Liang et al. [87]. Furthermore, α-tocopherols have been implicated in vitamin A metabolism [88], while cholesterol [89] and vitamin D metabolism share overlapping biosynthetic pathways [90].

Minerals are also present in hemp seeds, although their bioavailability is not well understood [7]. Minerals are classified as micronutrients due to their low dietary requirement (ranging from 1–2500 mg/day, depending on the mineral); they are nevertheless essential for various physiological and structural functions in human health. Minerals are categorized as macro-elements (required in amounts > 50 mg/day), including phosphorous (P), potassium (K), magnesium (Mg), calcium (Ca), and sodium (Na), and as micro-elements or in-trace elements (required in amounts of <50 mg/day), such as iron (Fe), manganese (Mn), copper (Cu), and zinc (Zn).

The mineral profile of hemp seeds has been relatively under-explored. However, reports indicate that the total mineral content ranges from approximately 4 to 7.6% [7,13,14]. The primary macro-elements are P, K, Mg, Ca, and Na, while the trace elements include Fe, Mn, Zn, and Cu [91]. Furthermore, authors have reported that the mineral composition of hemp seeds is influenced by environmental conditions, soil mineral composition, and plant variety [14].

Vitamins and minerals collectively contribute to the nutritional value of hemp seeds, supporting various aspects of human health.

**Table 1:** Hemp seed nutrients *.

Component	Concentration	Biological Effects and Properties	References
**Total Oil**	~25–35%		[14,39]
**Unsaturated fatty acids**	~90% of hemp seed total oil	Protective effects against cardiovascular diseases, obesity, diabetes mellitus, and anti-inflammatory disorders	[14,39]
**PUFAs**	~70–80% of unsaturated FAs		[10,14]
Linoleic acid (LA)	~55.1–63.7% of oil	Omega-6/omega-3 ratio, optimal value between 3:1 and 5:1 (EFSA) for reduction of chronic disease risk and mortality	[10,14,39,75]
α-linolenic acid (ALA)	~15.2–26.2% of oil	Neuroprotection, vasodilation of brain arteries, and neuroplasticity action	[14,39,75,92]
γ-linoleic acid (GLA)	~0.6–6.2% of oil	Anti-inflammatory action, reduction of deficit/hyperactivity disorder, cancer, dry eye syndrome, osteoporosis, diabetic neuropathy, ulcerative colitis, rheumatoid arthritis, and atopic dermatitis	[10,77,93,94]
Stearidonic acid (SDA)	~0.2–1.5% of oil	Sustainable omega-3 source, anti-inflammatory action	[10]
**Polysaccharides**	~20–30%	Prebiotic compounds, protection of intestinal epithelial cells from hydrogen peroxide-induced oxidative stress, and reduction of appetite and total LDL in hypercholesterolemia. Insulin sensitivity	[9,10,14,95,96]
Water-soluble polysaccharides	~5.5% of polysaccharides	Antimicrobial activity	[11,97]
Insoluble polysaccharides	~22% of polysaccharides	Decrease in obesity and diabetes mellitus	[11,14,97]
**Proteins**	~20–25% of total content	Antioxidant, antihypertensive, and hypo-allergenic agents	[25,39,41,57,66]
Edestin	~60–75% of total storage protein content	Vasodilatation and human blood circulation improvement; antioxidant and antihypertensive properties	[9,14,64,67]
Albumin	~25–30% of total storage protein content	High radical scavenging activity	[9,14,42,43]
Vicilin	~5% of total storage protein content	Solubility and foaming/emulsifying properties; radical scavenging activity	[9,14,42,43,46]
**Vitamin E** (Tocopherols)	~562.8–929.67 mg/kg	Mitigation of oxidative stress and prevention of degenerative diseases	[14,21,81,83]
γ -tocopherols	~92.5–93.3% of total tocopherols	Antioxidant, anti-inflammatory, and anticancer properties	[14,21,81,83,98]
δ-tocopherols	~1.9–3.5% of total tocopherols	Reduction of lipid accumulation in lipid storage disorders. Antiangiogenic effects	[14,21,81,83,99,100]
α-tocopherols	~3.8–6.2% of total tocopherols	Reduction of cardiovascular diseases, and iskemic stroke	[14,21,81,83,101]
**Vitamin A**	~78 mg/kg	Essential for healthy skin development Anti-inflammatory mechanism	[14,81,102]
**Minerals**	~4–7.6% of total content	Essential for human physiological and structural functions	[7,10,13,14,40,91]
Phosphorous (P)	~890–1.170 mg/100 g	Support for bone augmentation and maintenance	[10,103]
Potassium (K)	~250–2.821 mg/100 g	Blood pressure reduction and positive influence on the risk of stroke and coronary heart disease	[10,104]
Magnesium (Mg)	~237–694 mg/100 g	Support for nerve transmission, cardiac excitability, neuromuscular conduction, blood pressure, and glucose metabolism	[10,105]

* The values may vary depending on cultivars and cultivation conditions.

### 2.5. Bioactive Compounds

Hemp seeds secrete a wide variety of bioactive compounds, which are secondary metabolites produced by the plant in response to abiotic or biotic stresses [106] and play a pivotal role in plant-pathogen interactions [107]. They include terpenes, polyphenols, phytosterols, carotenoids, and phytocannabinoids families [7,106]. A list of the bioactive compounds found in hemp seeds is reported in Table 2.

Oil extracted from hemp seeds is characterized by a high concentration of terpenes, contributing to the flavor of cannabis-based foods [108]. Furthermore, hemp terpenes exhibit anti-inflammatory, anti-cancer, and antioxidant functions and have been shown to enhance the pharmacological benefits of CBD through the ‘entourage effect’ [109].

Hemp seeds are also rich in phenols, water-soluble compounds which are mainly contained in the hull fraction [16,40], often discarded during processing. These compounds, thanks to their chemical structure, have intrinsic antioxidant activity, and may protect cell constituents against oxidative damage, potentially limiting the risk of several degenerative diseases associated with oxidative stress. In fact, they show various physiological activities in the human body, including cardioprotective and anti-inflammatory effects [10].

The main phenolic compounds detected in hemp seeds are lignans, phenols derived from the shikimic acid biosynthetic pathway. Specifically, hydroxycinnamic acid amides (HCAAs) and lignanamides are present in relevant amounts, as reported by Leonard et al. [110].

Many phenolic compounds isolated from hemp seeds have demonstrated high radical scavenging activity compared with quercetin. Notably, the phenolic amide N-trans-feryoryltyramine and the lignanamides 3,3′-demethyl-grossamide and 3,3′-demethylheliotropamide also inhibited the acetylcholinesterase (AChE) enzyme. Therefore, they may have therapeutic potential for managing Alzheimer’s disease [111]. Moreover, Bourjot et al. [112] found that N-trans-caffeoyltyramine, among the phenolic amides extracted from hemp seeds, exhibited the highest antioxidant and arginase inhibitor activities, thus potentially benefiting conditions such as cancer, cardiovascular diseases, and fungal infections.

Recently, bioactive molecules belonging to the polyphenols (e.g., p-Coumaric acid 4-Hydroxybenzoic acid) and flavonoids (e.g., Glucosylvitexin and Vitexin-2-O-rhamnoside) families were identified [113]. The presence of these bioactive compounds may further explain the antioxidant activity and antimicrobial properties of hemp seeds. Relevantly, these metabolites persisted after in vitro digestion, significantly increasing the hemp radical scavenging capacity. Moreover, in addition to antioxidant properties, flavonoids show strong anti-inflammatory, anticancer, and cardioprotective activities [114].

Minor bioactive fat-soluble compounds, such as phytosterols, may also contribute to the health benefits of hemp seed oil by protecting against cardiovascular diseases [115]. Phytosterols, which have a cholesterol-like structure, can affect cholesterol solubility in the intestine, reducing its absorption [115]. Unlike cholesterol, phytosterols cannot be synthesized by the human body and are found exclusively in plants. Among the phytosterol components, β-sitosterol was most abundant, accounting for ~60–70% of the total phytosterol content [83].

Carotenoids are another group of fat-soluble bioactive compounds in hemp seed oil, which can act as antioxidants, potentially reducing the risk of degenerative diseases, like Parkinson and Alzheimer [87]. Among carotenoids, lutein was found to be the most abundant in hemp (1.4–3.4 mg/100 g of the whole hemp seed), followed by β-carotene and zeaxathin, whose contents strongly depend on genotype and genotype x growing year factors [53].

Hemp seeds contain only trace amounts of phytocannabinoids, with THC content well below the legal threshold of 0.3% and of 0.2% established in United States and Europe, respectively, making them safe for consumption [116]. The composition of these compounds varies among different hemp accessions, as reported in a recent study [12].

This diverse array of bioactive compounds contributes to the potential health-promoting properties of hemp seeds.

**Table 2:** Bioactive compounds in hemp seeds *.

Bioactive Compound	Concentration Values	Hemp Seed Part	Biological Effects and Properties	References
Phenolics			Antioxidant, anti-microbial, anti-inflammatory, anti-neuroinflammatory, neuroprotective, and anti-cancer action	[10,16,40,106,113]
Total phenolic content (TPC)	~100–300 mg GAE/100 g	Whole hemp seeds		[10,106,117,118]
Total lignanamides	~20–100 µg/g DW	Whole hemp seeds	Properties similar to the medicines used for the treatment of mild-to-moderate Alzheimer’s disease, such as galanthamine	[6,110,111]
Total hydroxycinnamic acid amides (HCAAs)	~22 CTE/g	Defatted hemp seeds	Antioxidant action	[106]
Phytosterols			Property against cardiovascular diseases, reduction of cholesterol absorption, antiviral, antifungal, and anti-inflammatory properties	[40,115]
Total phytosterol content (TPC)	~230 mg/100 g ~650 mg/100 g	Whole hemp seeds Oil		[119]
β-sitosterol	~140–160 mg/100 g ~390–455 mg/100 g	Whole hemp seeds Oil	Attenuation of epidermal hyperplasia and immune cell infiltration in the psoriasis-like mouse model	[83,119,120]
Terpenes			Anti-inflammatory, anti-cancer, and antioxidant functions	[109]
β-myrcene	~3170 ng/g ~1180 ng/g	Raw hemp seeds Roasted hemp seeds	Muscle relaxant and sedating effects	[109,121,122]
D-limonene	~1347 ng/g ~470 ng/g	Raw hemp seeds Roasted hemp seeds	Immunomodulatory properties, including antitumor effects	[121,123]
Carotenoids			Antioxidant activity	[87,124]
Lutein	~1.4–3.4 mg/100 g	Whole hemp seeds	Beneficial effects on eye health	[53,125]
β-carotene	~0.2–0.8 mg/100 g	Whole hemp seeds	Protection against skin damage	[53,126]
Zeaxanthin	~0.2–0.5 mg/100 g	Whole hemp seeds	Prevention of the progression of eye diseases and antioxidant protection of heart and skin	[53,127]
Cannabinoids	~2.3–234 mg/kg	Oil	Pharmacological benefits of CBD enhanced by terpenes, through the ‘entourage effect’	[85,109]

* The values can vary depending on the variety, cultivation conditions, and extraction method.

## 3. Hemp Seed By-Products

**Figure 2:**
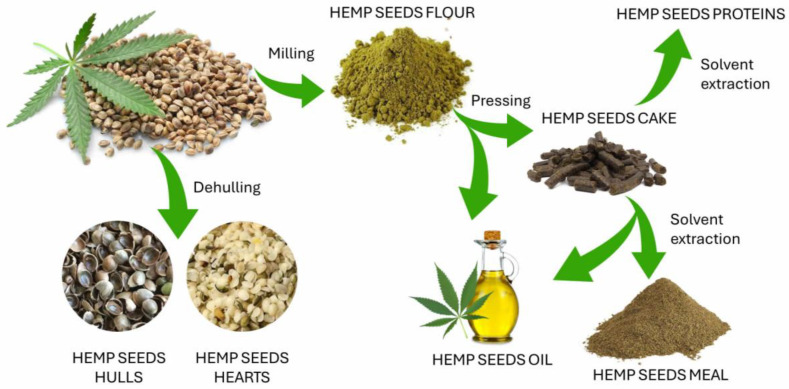
Schematic representation of the main products obtained from whole hemp seeds. Hemp seed hulls and hearts are obtained through the dehulling process. Whole hemp seeds are milled into hemp flour which is subsequently used to obtain hemp oil. The residues from these extractions include hemp seed cake (from pressing extraction) and hemp seed meal (from solvent extraction). Proteins are extracted from both cake and meal by-products [24].

There is a large variety of hemp seed by-products on the market, including whole hemp seed, dehulled hemp seed, hemp hulls, hemp flour, hemp seed oil, hemp seed cake, hemp seed meal, and hemp proteins [24]. Figure 2 illustrates the main processes to obtain these products from hemp seeds.

The dehulling process allows the obtainment of hemp seed hearts and hulls, which were often considered waste and discarded during oil extraction. However, an investigation carried out by Chen et al. [16] reevaluated the functional properties of hulls. Indeed, this study analyzed two hemp varieties, Bama and Yunma, to explore the relationships between TCP and free radical scavenging activities of hemp seed products (hearts and hulls), as well as to identify compounds with relevant antioxidant capacity. In particular, authors identified two compounds with predominant radical scavenging activity: N-trans-caffeoyltyramine and cannabisin B, suggesting that hemp seed hulls could be included in dietary supplements.

Hemp oil extraction processes and related techniques are crucial to preserve the nutritional composition of the seed oil [128]. Among the various methods, pressing and solvent extraction are the most used to obtain oils, with seed pre-treatment and processing conditions affecting their quality [8,128].

Techniques such as cold pressing, which involves mechanical management like extrusion or pressure without altering the oil’s characteristics, have been efficiently applied to extract hemp seed oils [129]. However, the extraction process cannot entirely prevent the presence of undesirable flavors resulting from oxidation [7,10]. Therefore, additional prevention strategies, such as storing hemp oil at low temperatures, are recommended to guarantee the stability of its nutritional quality and ensure its food and therapeutic functionalities [7,24].

Hemp flour, obtained from seeds through milling, is also an example of bio-sustainable raw material with a high nutritional value. It contains a greater content of bound phenols compared to free ones, which have well-known effects on cancer cell inhibition [22,130]. The most abundant free phenolic component found was cannaflavin C and the fundamental bound phenolic components were protocatechuic acid, caffeic acid, hydroxycinnamic acid, and cannaflavin C [22]. Furthermore, high levels in EAAs, gliadin, and glutenin-free protein profiles were also detected, thus making hemp flour appropriate for celiac disease foods.

A wide set of hemp protein concentrates (HPCs) and hemp protein isolates (HPIs) can be derived from solvent-extracted oil seed, and from pressed cake or meal [65]. The bioactivities HPIs, including antioxidant [45,65] and anti-inflammatory properties [81], underscore the potential of hemp seeds as protein source. Moreover, the amino acid content of proteins from these hemp seed by-products is significantly higher than that of whole hemp seeds [55].

## 4. Omics in Hemp Seeds

### 4.1. Genomics in Hemp Seeds

Recent advancements in sequencing technologies, ranging from short-read methods to third-generation long-read sequencing technologies such as single-molecule real-time (SMRT) sequencing (PacBio) and Oxford Nanopore Technologies (MinION) [131,132,133,134], have facilitated the creation of several hemp genome assemblies for different cultivars: ‘Purple Kush’ (‘PK’, a drug type Cannabis), ‘Finola’ (‘FN’; a fiber type Cannabis), ‘Jamaican Lion’ (‘JL’; a wild accession), and ‘CBDRx’ (‘cs10’; with high CBD content) [133,135]. Furthermore, a chromosome-level reference genome of the female seed hemp ‘yushe’, a Chinese cultivar was recently generated by integrating PacBio SMRT long reads and high-resolution chromosome conformation capture (Hi-C) mapping [136]. The genome size was approximately 783 Mb, with a heterozygosity rate of 1.95%. Furthermore, a whole-genome comparison of seed hemp ‘yushe’ with two other cultivars, wild hemp and marijuana, showed that ~14,440 gene families were shared among all three genomes and ~2635 were specific to hemp seeds [136]. The Gene Ontology (GO) enrichment analysis revealed that 30 genes were involved in the lipid catabolic process. The Kyoto Encyclopedia of Genes and Genomes (KEGG) enrichment analysis identified numerous genes involved in the oxidative phosphorylation, photosynthesis process, and in the proteasome. These findings may explain the abundance of antioxidant components and the unique biological effects associated with hemp seeds. Wei et al. [136] also detected approximately 35 genes involved in the lipid biosynthesis pathway, providing insights into the mechanisms of fatty acid biosynthesis in hemp seeds. Genes regulating early seed development included ~55 genes related to fatty acid synthesis, eight of which involved in the FA synthesis pathway. Furthermore, variations in the copy number (CN) of stearoyl-ACP desaturase (*SAD*) and fatty acid desaturase (FAD) genes were observed, and this could explain the variation in the oil content of hemp seed. Finally, two genes (*Csa.DXS2* and *Csa.HPPD1*) resulted involved in the vitamin E biosynthesis pathway.

The availability of hemp genomic information, combined with advancements in sequencing technologies, has also facilitated studies on the genetic basis of complex hemp traits. Furthermore, whole-genome sequencing has enabled the mapping of quantitative trait loci (QTL), associated with variations in numerous agronomic and biochemical traits [137].

Advances in hemp genomics and sequencing technologies have also simplified genome-wide association studies (GWAS) to investigate the genetic basis of various traits in hemp. GWAS in hemp have been used for various purposes, including an exploration of flowering time and sex information using a panel of accessions grown in different European locations and a large set of single-nucleotide polymorphism (SNP) markers [138]. To the best of our knowledge, GWAS studies aimed at identifying traits and candidate genes associated with plant seed nutrient composition, such as oil and protein content, have been conducted in several plant species [139,140], but not yet in hemp. This represents a notable gap, especially considering that the nutritional quality of hemp seeds, including protein quality, as determined by amino acid composition and digestibility, is influenced by several factors. Genotypic variability and agronomic conditions, such as soil fertility and post-harvest processing, can significantly affect the amino acid profile and the relative proportions of seed components [141]. Therefore, applying GWAS approaches to hemp, including those focused on seed yield and quality traits [137], could provide valuable insight into the genetic basis of its nutritional properties and support for targeted breeding strategies.

Genomic Selection (GS), which often builds upon findings from GWAS, such as relevant genetic markers or SNPs, is used to predict genomic estimated breeding values [142,143]. While GS has been applied in cannabis studies [144,145], these efforts have primarily focused on understanding of the evolutionary history of *C. sativa* and optimizing cannabinoid profiles, rather than directly targeting nutritional traits. The limited application of GWAS and GS to investigate the nutritional composition of hemp seeds may be attributed to the relatively recent recognition of hemp as a valuable food resource. Nevertheless, these existing studies provide a foundational framework for future research aimed at improving the nutritional quality of hemp.

Moreover, the recent development of genome editing technologies, such as clustered regularly interspaced short palindromic repeats (CRISPRs), offers promising opportunities to improve hemp varieties with greater precision. Gene-editing tools have already enabled the target modification of genes involved in the biosynthesis of cannabinoids, flavonoids, carotenoids, and seed oil quality [146,147,148]. These advances form the bases for the development of hemp cultivar with enhanced nutritional and functional properties.

In addition, the role of the plant microbiome in influencing the accumulation of secondary metabolites has gained increasing attention. Advances in metagenomic techniques, particularly those based on 16S rRNA sequencing [149], have revealed the significant impact of microbial communities on plant fitness and the elicitation of secondary metabolite production [150].

Regarding genomic studies on hemp seeds, we identified two particularly relevant works [64,67], which are discussed in Section 4.2, as they also incorporate transcriptomic approaches.

### 4.2. Transcriptomics in Hemp Seeds

RNA sequencing (RNA-seq) is the most widely used transcriptomic technique, primarily employed to identify differentially expressed genes, elucidate regulatory networks, and derive biological insights from gene expression data. This method has been applied in several hemp seed studies [47,151]. Quantitative reverse transcription polymerase chain reaction (qRT-PCR), a molecular biology technique used to quantify gene expression and validate RNA-seq results, has also been adopted in hemp seed investigations [64,67,76].

Transcriptomic analyses of hemp seeds have focused on various aspects, including the biosynthesis of PUFA, seed storage proteins—mainly 11S edestin, 2S albumin, and one 7S vicilin-like—and bioactive compounds, such as flavonoids.

Several studies focused on Bama hemp, cultivated in the Bama region of Guangxi, China, whose seeds are abundant in unsaturated FAs, particularly ω-6 and ω-3. For instance, Nie et al. [76] applied a combined analysis of metabolomic and transcriptomic data to investigate the fatty acid formation patterns in Bama hemp seeds during their development stages. They found that the peak period of nutrient accumulation occurs during the mid-stage rather than in the late development stage. Furthermore, the metabolomics analysis showed that seed oil accumulation is positively correlated with seed size and sugar, protein, and starch content. Finally, transcriptomics analysis identified key genes involved in the metabolic pathway of linoleic, α-linolenic, and jasmonic acid, such as the FAD2 gene, which were found to be highly upregulated.

Hemp seed proteins were investigated in a comprehensive transcriptomics study [64], which involved hemp inbred lines. This study identified and characterized gene families encoding precursor polypeptides of 11S edestin, 2S albumin, and 7S vicilin-like. Using a genome-wide identification approach, all edestins were linked to specific DNA fragments. Nucleotide sequences were analyzed using BLAST or tBLASTn search programs (https://blast.ncbi.nlm.nih.gov/Blast.cgi (accessed on 17 May 2025)) to find homologous genes, while multiple alignments were made using Clustal Omega tool (http://www.ebi.ac.uk/Tools (accessed on 17 May 2025)). All three protein types were very rich in arginine and glutamic acid, with type3 edestin also abundant in cysteine and methionine. Six edestin genes and two 2S albumin genes were also isolated, while only one 7S-vicilin like gene was identified. A gene expression analysis (performed by qRT-PCR) also revealed that all genes are expressed in the seed, with edestin type1 and 2S albumin having the highest expression, whereas 7S vicilin-like genes showed the lowest expression.

Furthermore, in a study by Docimo et al. [67], seven cDNAs encoding edestin were isolated from the *C. sativa* variety Carmagnola. Based on sequence similarity, four edestin genes were classified as edestin type1 (*CsEde1A*, *CsEde1B*, *CsEde1C*, and *CsEde1D*) and three as edestin type2 forms (*CsEde2A*, *CsEde2B*, and *CsEde2C*). The qRT-PCR analysis revealed that both edestin types are expressed in hemp seeds during their development, exhibiting a high percentage of arginine (~12%), with edestin type2 particularly rich in methionine residues (~2.35%) when compared with edestin type1 (~0.80%). A proteomics analysis determined the amino acid composition in *CsEde1* and *CsEde2* types, suggesting that these proteins could be used to improve the nutritional quality of food products.

The overall expression pattern of genes and metabolite accumulation were also investigated during different hemp seed developmental stages [151]. In this study, the metabolomic analysis of hemp seeds from the Bama region revealed a higher number of flavonoids compared to other Chinese varieties, particularly metabolites such as cannflavins (A, B, and C), trigonelline, citric acid, vitexin, choline alfoscerate, and choline, which may contribute to the longevity of the local population. Furthermore, a metabolomics analysis combined with a transcriptomic approach across four hemp seed maturity stages showed a gradual decrease in the overall gene expression pattern and metabolite accumulation during seed development, and underlined the role of transcription factor genes, such as MYB, NAC, and GRAS, in the regulation of these metabolites.

### 4.3. Metabolomics in Hemp Seeds

Metabolomics approaches have been extensively applied to investigate the nutrient and bioactive compound profiles of hemp seeds. One of the most comprehensive metabolomic studies to date is that of Ning et al. [12], which identified over 1000 metabolites across various hemp seed cultivars. These included approximately 200 flavonoids, 85 alkaloids, 150 phenolic acids, and a range of unsaturated fatty acids—particularly linoleic acid (LA) and α-linolenic acid (ALA)—present in an optimal ratio of ~2.5–3:1, which is considered beneficial for cardiovascular health.

The most commonly used platforms for metabolite profiling are gas chromatography (GC) and liquid chromatography (LC), both typically coupled with mass spectrometry (MS). LC is preferred for non-volatile compounds, while GC is suited for thermally stable molecules. High-performance liquid chromatography (HPLC) has also been widely used.

Additionally, nuclear magnetic resonance (NMR) spectroscopy has been used for metabolite detection in complex matrices such as hemp seeds [152]. Although NMR offers structural insights without destructive sample preparation, it has been underutilized in hemp seed metabolomics, mainly applied for oil quality assessment [153]. Given the complexity of the hemp seed metabolome, combining multiple analytical techniques provides the most comprehensive results.

Since 2014, omics studies have explored the composition of hemp seed oil. For instance, Porto et al. [52] carried out metabolomics studies on four industrial hemp cultivars, Felina 32, Chamaeleon, Uso31, and Finola, and found that all cultivars were high in ω-6 and ω-3 compounds, with high oxidation stability and an acceptable quality. These properties were also reported in other hemp seeds varieties, such as the Italian Carmaenecta, Enectaliana, and Enectarol [154].

Chen et al. [50] studied hemp seeds’ lipid profile in seven villages in Bama region by using a targeted metabolomics approach. More in detail, a total of approximately 1020 metabolites, including around 135 lipids, were identified, and an abundant unsaturated fatty acid content was also detected. These findings confirmed the high nutritional and therapeutic value of hemp seeds, particularly in those from the Bama region.

About polysaccharides, only recently, their comprehensive analysis has been conducted [155], with seeds from 20 hemp cultivars that were analyzed for a series of traits, including complex carbohydrates. A metabolomic analysis revealed different polysaccharides in the heart seed fraction (dehulled seed) and in the hull fraction. The heart fraction mainly contained xyloglucan and pectin, while cellulose and xylan were predominant in the hulls. The overall monosaccharide content in the heart was lower than in the hulls, where the xylose was the most relevant non-cellulosic polysaccharides, ranging from approximately 5.5 to 17% *w*/*w* among the different hemp cultivars. Sucrose was the most abundant sugar in both hull and heart hemp seeds, although it was less abundant in hulls than in hearts. Furthermore, the heart fraction contained few starch granules (<2%).

With the aim to extend the use of hemp seed polysaccharides to functional applications in the food industry, another study [11] investigated the physicochemical characteristics of soluble hemp seed polysaccharides using the HPLC technique, comparing them with those of flax seeds. The study evaluated molecular weight distribution, shape conformation, total sugar content, and emulsification properties. The results demonstrated that hemp seeds and flax seeds soluble polysaccharides displayed a great emulsion stability (90% at the end of the 21st day of storage), suggesting they may represent a promising food emulsifying agent.

Hemp seed bioactive compounds were also largely studied with metabolomics approaches. Ning et al. [12], employed a targeted metabolomics approach based on an ultrahigh-performance liquid chromatography method coupled with a triple quadrupole mass spectrometry system (UHPLC-QQQ-MS/MS) to study several metabolites, including flavonoids, alkaloids, and phenolic acids, in seven hemp seeds varieties with relevant phenotypic differences. The specific metabolite characteristics and the hub metabolites of each variety were investigated by using a weighted gene co-expression network analysis (WGCNA) approach, suggesting that the content of the metabolites might be responsible for their differences. Furthermore, the proportions of each class of metabolites were similar in all varieties; of these, lipids, flavonoids, and phenolic acids were the major metabolites.

In another study [106], the optimization of phenolic compounds extraction from defatted hemp seeds using a simplex lattice mixture containing water, methanol, and acetone was achieved. The profile was then analyzed by high-performance liquid chromatography equipped with photodiode array detection tandem electrospray ionization mass spectrometry (HPLC-DAD/ESI-MS/MS) techniques, and the results confirmed the predominance of hydroxycinnamic acid amides and lignanamides, as already reported by Leonard et al. [110]. This distinctive phenolic profile of hemp seed was also confirmed in a recent metabolomics study [156], whose results highlighted the high variability of bioactive metabolites across different hemp seed accessions.

Izzo et al. [85] investigated carotenoids and polyphenols, demonstrating that both bioactive compounds contribute to the oxidative stability of hemp seed oils by enhancing resistance to photo-oxidation.

About terpenes, Jeong et al. [157] used electronic sensors and gas chromatography (GC–MS/Olfactometry GC–MS) to analyze the flavor of six types of oil extracted from roasted hemp seeds under various conditions and identified α-pinene, β-pinene, sabinene, α-phellandrene, linalool, and 1,3,8-*p*-menthatriene as the most abundant terpenes. The roasting technique, often used in oil processing to improve the extraction yield and sensory characteristics, was also applied by Mansouri et al. [121]. Indeed, using metabolomics techniques, such as solid-phase microextraction (SPME) and GC, the study found that volatile compounds in unroasted hemp seeds were dominated by terpenes (~85%), and after roasting, they still constituted more than 69% of the identified volatile compounds, with β-myrcene and D-limonene being predominant in both cases.

A comprehensive qualitative and quantitative analysis of phytosterols was performed by Blasi et al. [83], which, by using a high-resolution gas chromatography (HRGC) method, revealed that β-sitosterol was predominant in hemp seed oil, followed by campesterol.

The complete cannabinoid profile of ten commercially hemp seed oils using an untargeted metabolomics approach based on a LC method coupled with HRGC, showed the presence of 32 phytocannabinoids, including CBD, THC, cannabicromene (CBC), and cannabinol (CBN) [158]. Finally, Izzo et al. [85], in accordance with Citti et al. [158], reported a cannabinoids concentration ranging from 2.265 to 233.8 mg/kg across different hemp seed oils.

Metabolomics studies were also carried out on hemp seeds by-products. They have confirmed the nutritional value of the hemp oil by-products, mainly pressed cake and meal. For instance, Occhiuto et al. [23] used a metabolomics analysis to study cold-pressed hemp seed oil and hemp seed meal, demonstrating not only the well-known properties of the oil, such as high content of total phenols, flavonoids, and tocopherols, but also many benefits of the hemp seed meal. The results indicated that part of the polyphenols and tocopherols are retained, preserving the ω-6/ω-3 fatty acids ratio identified in the hemp seed oil. This indicates a very relevant nutritional profile, rich in proteins and crude fibers, thus suggesting its potential re-use as functional food.

In hemp flour, Buzzanca and Di Stefano [22] used UHPLC-ESI/Q-TOF-MS techniques to show a greater content of bound phenols compared to free ones. The most abundant free phenolic component found was cannaflavin C and the fundamental bound phenolic components were protocatechuic acid, caffeic acid, hydroxycinnamic acid, and cannaflavin C. Furthermore, high levels of EAAs (~16 g/100 g), gliadin, and glutenin-free protein profiles were also detected. Interestingly, Sciacca et al. [44] performed a study, based on the use combined metabolomics techniques (GC-MS, HPLC-FLD, HPLC-FLD, and GC-FID) to explore the effects of incorporating different percentages of hemp seed flour into fortified bread, evaluating the impact on organoleptic characteristics, total polyphenols, free radicals scavenging activity, and amino acid content. In detail, by replacing various percentages of durum wheat semolina with hemp seed flour, the researchers found that the fortified bread exhibited increased antiradical and antioxidant activity, along with enhanced amino acid content, in terms of glutamic acid, tyrosine, proline, and essential amino acids. Interestingly, the sensory analysis confirmed a high acceptability of this nutrient-rich bread.

Many metabolomics studies used combined analytic techniques, particularly in the analysis of lipids [50,154], polyphenolics [12,106], carotenoids [85], and terpenes [157]. This integrative approach has also been applied to the study of hemp seeds by-products [22,23,44], helping to overcome the limitation of individual techniques and providing more comprehensive and accurate results.

Furthermore, phytosterols were studied using GC high-resolution techniques [83,158]. In fact, phytosterols exist in complex plant matrices, often alongside other compounds that can interfere with their extraction and analysis. Therefore, the presence of these components needs specialized extraction and analysis methods to accurately determine phytosterols. Finally, combined and GC high-resolution techniques were applied for exploring cannabinoids, due to their complex matrices.

### 4.4. Lipidomics in Hemp Seeds

Lipidomics, a relatively recent omics discipline introduced in the early 2000s [159,160], enables the comprehensive profiling of the lipidome in plants and seeds, including hemp. Its growing relevance reflects the increasing importance of lipid-based products in nutraceutical, food, and clinical applications [161]. Despite the recognized richness of hemp seeds in bioactive polyunsaturated fatty acids, their complete polar lipidome remains largely unexplored [162].

Mass spectrometry (MS) is the method of choice for lipidomics, due to its high sensitivity and specificity [163]. Techniques such as LC-MS, GC-MS, and Q-TOF are commonly used, along with ‘shotgun lipidomics’, a direct-infusion ESI-MS approach that enhances the detection of specific lipid classes [164]. High-resolution MS (HRMS) further improves compound identification in complex samples and has been applied in several hemp seed lipidomics studies [161,162,165,166,167]. For example, Buré et al. [161] investigated the phospholipid (PL) composition of oilseed cakes, including hemp, to support by-product valorization. PLs, which represent ~0.6–2% of hemp seed lipids [168], are essential for membrane structure and cellular regulation [169]. The study combined three MS-based methods—shotgun, reverse-phase LC (RPLC), and normal-phase LC (NPLC)—to obtain complementary data: shotgun for PL identification, RPLC-MRM for detecting minor species, and NPLC for identifying lysophospholipids and cardiolipins.

Cerrato et al. [165] developed a protocol and analytical workflow for the determination of polar lipids in hemp seeds. The method involved lipid extraction, isolation using graphitized carbon black sorbent, and analysis via liquid chromatography (LC) coupled with high-resolution mass spectrometry (HRMS). Using Lipostar software (https://www.moldiscovery.com/software/lipostar/ (accessed on 17 May 2025)), ~190 polar lipids—including sulfolipids and phospholipids—were identified. In a subsequent study, Cerrato et al. [162] characterized lipid extracts from nine hemp seed varieties using an untargeted HRMS-based platform. Compound Discoverer software (https://mycompounddiscoverer.com/ (accessed on 17 May 2025)) enabled the detailed annotation of 184 lipid species, including 26 fatty acids and 158 phospholipids, such as N-acylphosphatidylethanolamines. The study also provided insights into the regiochemistry of free and conjugated fatty acids. Kozub et al. [167] used LC-QTOF-MS to profile diacylglycerols (DAGs) and triacylglycerols (TAGs) in cold-pressed oils from camelina, flax, and hemp seeds. They identified 36 DAGs and 105 TAGs in hemp oil and proposed 27 lipidomic markers for oil authenticity testing.

Bakhytkyzy et al. [166] established a micro-solid phase extraction platform combined with HRMS to annotate and quantify over 60 lipids in hemp seed oil. Identified classes included LPC, LPE, PC, PE, DG, and TG. A multivariate analysis showed strong lipidomic similarities among flax, hemp, and chia seeds, with ALA and LA present across all the lipid classes. Together, these studies underscore the growing potential of lipidomics to deepen our understanding of hemp seed lipid composition, support the valorization of by-products, and guide the development of nutritionally enhanced cultivars and functional food applications.

### 4.5. Ionomics in Hemp Seeds

One of the earliest studies exploring the minerals composition of hemp seeds was conducted by Callaway et al. [6]. However, to date, the mineral profile of hemp seeds remains relatively underexplored, particularly through omics approaches such as ionomics. Elemental analysis methods typically rely on the electronic properties of atoms [170]. Among these, inductively coupled plasma (ICP)-based techniques, such as ICP optical emission spectroscopy (ICP-OES) or mass spectrometry (ICP-MS), are widely employed. ICP generates a high-temperature plasma in which atoms are ionized, enabling sensitive detection [170]. While ICP-OES is effective for quantifying major elements, it is generally less sensitive than ICP-MS [170]. The flame atomic absorption spectroscopy (AAS), another technique used in hemp seed ionome studies [171], also relies on atomic electronic transitions; however, it is is slower and less sensitive compared to ICP-based methods [172].

Lan et al. [173] employed ionomics (inductively coupled plasma coupled to optical emission spectrometry—ICP-OES) to explore how growing conditions affect the seed composition and particularly the mineral composition of industrial hemp varieties. Their findings revealed significant variations in the levels of Ca, Na, K, Fe, and Mn across different hemp varieties, while P and Mg remained consistent. By metabolomics techniques, oils were also analyzed and results showed that most of the hemp seeds varieties differed significantly for the fatty acid profile.

In a separate study, Siano et al. [40] evaluated the mineral composition of ‘Fedora’ seeds using ICP-OES. Their results indicated higher concentrations of Fe, followed by Zn, Mn, and Cu, while molybdenum (Mo), nickel (Ni), and cobalt (Co) were present in minor amounts. The study, by metabolomics approaches, also explored other hemp seed nutrients, such as fatty acids, phytosterols, and phenols. The fatty acid ω-3/ω-6 fraction is in agreement with the nutritionally optimal 3/1 ratio. β-sitosterol and other phytosterols sterols dominated the unsaponifiable fraction. Hemp seeds, flour, and oil contained 767 ± 41, 744 ± 29, and 21 ± 5 mg GAE/Kg total polyphenols, respectively.

Recently, Esteban et al. [171] determined the mineral and antinutrient (phytates) contents of several varieties of whole and hulled hemp seeds. AAS was used for mineral element analysis. By using UV/visible spectroscopy techniques, the study showed that P was the most abundant mineral component with higher values in hulled seeds (1.1 g/100 g) compared to whole seeds. Conversely, K, Mg, Zn, Ca, Mn, and Cu amounts were prevalent in whole seeds. However, the study also highlighted that the presence of phytates, which were abundant in hulled hemp seeds, could reduce mineral bio-accessibility, compromising P, Fe, and Zn absorption.

Furthermore, Ramos-Sanchez et al. [174] characterized a large set of hemp seed products and by-products, such as hemp seed oil, dehulled seeds, proteins, and cake, at macro- and micro-nutrient and bio-active levels. Focusing on the quantification of the micro-element content, the analysis was performed by the ICP-MS technique, and it was found that all the investigated products were promising sources of micro-nutrients like potassium, magnesium, and phosphorus. These results indicate that the manufacturing processes of the hemp seeds into hemp seed-based samples do not affect the mineral content profile. This implies that factors including the type of raw material (whole seeds or hemp seed hearts) and the temperature used during line processing do not lead to substantial alterations in the micronutrient composition.

### 4.6. Proteomics in Hemp Seeds

Hemp seed proteome was initially investigated only in the Korean cultivar Cheungsam [66], where the use of conventional techniques, such as 2-D electrophoresis, MS, and search in the nr-NCBI protein database allowed the identification of 168 unique protein spots. However, only one was assigned to *C. sativa*, while most of them were referred to other plants. Aiello et al. [41] performed on the seeds of the French cultivar “Futura” further proteomic analysis extending the initial *C. sativa* proteome, identifying in total 181 expressed proteins. Combinatorial peptide ligand libraries (CPLLs) were used for protein equalization [175]. Subsequently, Mamone et al. [176] investigated the proteome of the Italian variety “Carmagnola”, identifying 6 and 30 proteins by searching the *C. sativa* and *Arabidopsis thaliana* database. In addition, they showed that only 12 of the protein fragments were conserved after the in vitro brush border membrane digestion of hemp protein isolate. Several other studies were carried out on the hemp seed proteome, enriching the previous knowledge with new evidence [46], and also exploring the influence of several factors, such as the extraction methods [177] and the environments conditions [42,43].

In recent years, several proteomic techniques have been employed to characterize the protein composition of hemp seeds. The most commonly used protein extraction and separation techniques include sodium dodecyl sulfate–polyacrylamide gel electrophoresis (SDS-PAGE), which separates proteins based on molecular weight, and two-dimensional gel electrophoresis (2D-GE), which resolves proteins by both isoelectric point (pI) and molecular size. Both techniques have been widely applied on several hemp seeds [42,43,45,55,178].

For protein identification and quantification, mass spectrometry (MS) remains the gold standard. Techniques such as matrix-assisted laser desorption/ionization time-of-flight (MALDI-TOF) MS, suitable for peptide mass fingerprinting, and electrospray ionization quadrupole time-of-flight (ESI-QTOF) MS, ideal for peptides/proteins sequencing, have been effectively used in hemp seed proteomics [42,43]. These are often coupled with LC forming LC-MS or LC-MS/MS platforms, which enhance separation and detection sensitivity [41,46].

Size exclusion chromatography—high-performance liquid chromatography (SEC-HPLC) has also been employed in hemp seeds proteomics [55], offering high-resolution, rapid, and reproducible separation based on molecular size. While HPLC/UV/RI (refractive index) is not a core proteomics tool, it is valuable for protein purification and quantification. Similarly, multi-angle light scattering (MALS) is primarily used for protein biophysical characterization rather than protein identification. A notable study by Zha et al. [179] applied a combined SEC-HPLC-UV/RI/MALS approach to characterize hemp protein isolates (HPIs), demonstrating the utility of this integrated method.

More recently, shotgun proteomics has emerged as a powerful, high-throughput tool to explore hemp seed proteome [176,177,180]. Unlike top-down approaches that analyze intact proteins, shotgun proteomics involves enzymatic digestion into peptides, followed by LC-MS/MS analysis. This method enables the identification and quantification of thousands of proteins in a single run and is particularly effective for characterizing hydrophobic and low-abundance proteins.

Many proteomics studies investigated the hemp seed protein profile, and confirmed that, like the other components of hemp seeds, it is affected by environmental conditions. For instance, in Cattaneo et al. [42], the impact of mountain environments on the protein content of two hemp cultivars (Finola and Futura 75) seeds was investigated. Using a combination of analytical techniques, including SDS-PAGE, 2D-gel electrophoresis, and mass spectrometry-based methods, the study demonstrated that mountain environments have a more pronounced influence on the protein profile of Finola seeds. Furthermore, Finola seeds of plants cultivated in mountain conditions exhibited a higher overall protein content compared to those coming from plants grown in the experimental area. Subsequently, Cattaneo et al. [43] investigated the impact of hemp varieties and environments on seed protein profiles and antioxidant activity. Utilizing MALDI-TOF mass spectrometry, the study proved the power of this technique in discriminating between hemp cultivars. Furthermore, a detailed fingerprinting analysis revealed that proteins coming from seeds collected in the mountain site showed a higher radical scavenging activity and higher levels of lower molecular weight compounds compared to reference seeds.

Aiello et al. [41], through a protein–protein association network, correlated hemp seed proteins previously identified with SDS-PAGE and nLC-ESI-MS/MS techniques, thus providing a comprehensive proteome characterization.

A recent study [46] investigated the proteome of whole-seed, dehulled seed, and hull on two cultivars, Santhica 27 and Uso-31, by using LC-MS/MS techniques. More specifically, seed storage proteins were the most abundant class, with averages of approximately 65%, 71%, and 57% for whole seeds, dehulled seeds, and hulls, respectively. Edestins were the most abundant proteins, followed by vicilin-like proteins and albumins. Furthermore, proteins related to defense and stress responses were more abundant in hulls than in dehulled seeds, demonstrating their potential as a protein source of interest.

The two most abundant HPI fractions, water-soluble albumin and salt-soluble globulin, have been extensively studied through proteomics-based studies. Malomo and Aluko [181] utilized defatted hemp seed meal to extract these proteins, and assessed their structural and functional properties. Their findings showed that albumin exhibited significantly higher protein solubility than globulin, while both proteins demonstrated similar emulsion forming ability.

Mamone et al. [176] investigated the HPI allergenicity, aware of the fact that the introduction of hemp-based products, such as HPI, in the diet of some individuals, could lead to a sensitization to plant-derived allergens. They showed that all known hemp allergens, including the major thaumatin-like protein and LTP, were entirely eliminated by the HPI production process. Previous allergens of hemp have already been reported [182], including nsLTP/PR-14 (Can s 3, 10 kDa); profilin (14 kDa); oxygen-evolving enhancer protein (23 kDa); TLP/PR-5 (38 kDa); and ribulose-1,5-biphosphate carboxylase/oxygenase (Can s RuBisCo, 50 kDa). However, of these allergenic proteins, only RuBisCo has been detected in hemp seeds [41,66]. Therefore, all these data support the use of HPI as an ingredient for hypoallergenic foods.

A series of studies exploiting shotgun proteomics also proved that different extraction and/or dehydration methods can strongly affect hemp seed proteomes, with micellisation yielding more albumin, oleosin, and sulphur amino acids than alkaline extraction, and dehydration reducing the retention of allergenic proteins [177].

Other omics studies investigated how extraction methods influence HPI and HPC, focusing on yield, solubility, and biological functionality with the aim to find efficient extraction processes able to optimize these parameters and boost the recovery of protein fractions rich in essential amino acids. The dehulling process has an impact on composition and functional characteristics of HPI. This technique significantly increases the HPI recovery yield, also resulting into a major accumulation of terpenes. However, dehulled HPIs show a lower thermal stability than non-dehulled HPIs, as demonstrated by means of SDS-PAGE, and other techniques suitable to characterize proteins, such as SEC combined HPLC/UV/RI and MALS [55].

It was demonstrated that a specific mechano-chemical process, based on the use of the ball milling method, improves the solubility of proteins [183]. By using this grinding method, which generates localized high pressure, it was also shown that the careful adjustment of extraction parameters can significantly increase yield and optimize protein functionality [183].

Moreover, different solvents may have an impact on proteins extracted from hemp seed meal. In fact, the test of several aqueous NaOH, KOH, NaHCO_3_, and NaCl solvents, at different concentrations, has demonstrated that, while alkali-based solvents provide a higher extraction yield, extraction with water provides the highest proportion of proteins containing essential amino acid [178].

Further advancements were made by Cabral et al. [45], who investigated the effect of extraction methods on HPI from defatted hemp seed meal. They analyzed techniques based on high-pressure processing (HPP) pre-treatments combined with conventional or ultrasound-assisted extraction (UAE) methods. The study revealed that the maximum protein recovery (~62%) and the highest protein purity (~75%) were achieved with HPP (200 MPa) and UAE. Notably, the UAE method improved the extraction of all amino acids compared to the conventional extraction method, independently from HPP pre-treatments. Furthermore, ultrasound-based protein extraction methods are identified as a green and efficient alternative to conventional methods [184].

## 5. Multi-Omics in Hemp Seeds

Next-generation sequencing (NGS) technologies have revolutionized plant biology by enabling the elucidation of complex molecular processes in crops known for their nutritional and therapeutical properties [185]. Building on this, integrated multi-omics approaches, combining genomics, transcriptomics, proteomics, metabolomics/lipidomics, ionomics, and other omics layers have been developed across a wide range of crop species. These approaches allow for the discovery of synergies among heterogeneous datasets, offering a holistic view that overcomes the limitations of single omics studies. By integrating data across biological levels, multi-omics enhances our understanding of molecular mechanisms, improves predictive power for complex traits, and reveals regulatory networks underlying key agronomic, nutritional, and therapeutic traits [186]. The increasing availability of high-throughput technologies has facilitated the generation, integration, and interpretation of large-scale multi-omics datasets. In parallel, the development of advanced computational algorithms has enabled the reconstruction of complex regulatory networks and the identification of biomarkers, accelerating research and applications in plant science [187].

In hemp, multi-omics approaches have enabled comprehensive hemp genetic and metabolic mapping, detailed characterization and exploration of sub-species relationships, genotypes and phenotypes associations, and the discovery of novel metabolites and biomarkers relevant to breeding programs [36]. However, many of these studies have applied omics techniques in parallel rather than through fully integrated data models. While this strategy has yielded valuable insights, true multi-omics integration—requiring sophisticated bioinformatics pipelines—remains a challenge [187,188]. However, discoveries have been made through the analysis of each omics dataset individually and then comparing the results to find for correlations.

Several studies exemplify the power of multi-omics in hemp seed research. For instance: (i) Nie et al. [76] combined transcriptomics and metabolomics to identify key genes and metabolic pathways involved in fatty acids biosynthesis; (ii) Docimo et al. [67] integrated genomic, transcriptomic, and proteomic data to elucidate the primary structure and gene expression of health-promoting seed proteins, laying the groundwork for future bioactive peptide research; (iii) Ponzoni et al. [64], similarly combined genomic and transcriptomic data to provide a detailed analysis of hemp seed proteins; and (iv) Duan et al. [151], used transcriptomic and metabolomic integration to highlight the role of transcription factor genes, such as MYB, NAC, and GRAS, in the regulation of metabolites accumulation during hemp seed development.

Beyond plant biology, multi-omics approaches have also been applied to explore the clinical potential of hemp seeds. For example, Lu et al. [78] used metabolomics and 16S rRNA gene sequencing to demonstrate that Bama hemp seed oil modulates aging-related biomarkers and gut microbiota composition in rats. Furthermore, Yu et al. [47] combined metabolomics, proteomics, and transcriptomics to show the therapeutic potential of hemp oil in colorectal cancer. Meanwhile, Gong et al. [189] integrated transcriptomic and metabolomic data to uncover the protective effects of hemp oil against non-alcoholic steatohepatitis in mice.

These studies underscore the value of multi-omics in providing more comprehensive and reliable insights than single-omics approaches. Table 3 summarizes omics studies focused on nutrients and bioactive compounds in hemp seeds, while Table 4 presents those conducted on hemp seed by-products.

## 6. Conclusions

This review highlights the growing body of omics-based research focused on the nutritional and bioactive components of hemp seeds and their bio-products. Among the various omics disciplines, metabolomics emerged as the most widely applied approach. A range of metabolomics techniques, selected based on the specific class of compounds under investigation, has significantly advanced both the qualitative and quantitative profiling of hemp seed composition. These methods, along with weighted gene correlation network analysis, have been instrumental in identifying key metabolites. Despite these advances, notable gaps remain. The polar lipidome of hemp seeds is still largely unexplored, even though these seeds are known to be rich in biologically active polyunsaturated fatty acids—a key area of interest for lipidomics. Similarly, the mineral profile of hemp seeds has received limited attention, particularly through omics approaches such as ionomics.

Proteomics, on the other hand, has seen substantial development. Given the increasing industrial interest and the changes of legal restrictions on the cultivation of low-THC cannabis cultivars in several countries, updates to the proteomic database for hemp seed are anticipated as research progresses in the upcoming years. Numerous studies have demonstrated its effectiveness in profiling thousands of proteins with varying abundance in hemp seed samples. The proteomics findings discussed in this review revealed significant quantities of proteins, including bioactive and functional peptides, present in hemp seeds both pre- and post-processing. The chemistry and quality characteristics of these proteins make them suitable for applications in food and in feed industries.

Genomics has also contributed to our understanding of hemp seed biology, particularly in elucidating molecular pathways involved in the biosynthesis of key compounds. However, a critical gap remains: GWAS and GS approaches targeting specific seed traits—such as oil composition or protein content—are still lacking. These tools could greatly enhance our understanding of the genetic basis of these traits. In transcriptomics, the literature is similarly sparse. Only a few studies have investigated gene expression regulation during seed development, a crucial aspect for improving seed yield and quality.

Multi-omics approaches that integrate genomics, metabolomics, proteomics, and transcriptomics have demonstrated the potential of hemp seeds in both nutraceutical and medical contexts. However, the studies conducted so far often lack true data integration and do not employ systems biology or network modelling approaches. The application of such integrative frameworks could significantly enhance our ability to model and predict the interactions among genes, proteins, and metabolites, ultimately deepening our understanding of their roles in human health.

This review aims to provide a comprehensive resource for researchers exploring the hemp seed transcriptome, metabolome/lipidome, ionome, and proteome. By advancing the understanding and utilization of hemp seeds and their by-products in food, feed, and medical applications, we hope to contribute to positioning hemp as a sustainable and valuable resource for the future.

## Figures and Tables

**Table 3:** Relevant omics studies on nutrients and bioactive compounds in hemp seeds, grouped according to the omics approach.

Nutrients and Bioactive Compounds	Omics Approach	Hemp Variety	Main Methods	Main Results	References
PUFAs, tocopherol crude proteins, and phenolics	Metabolomics	CanMa, Anka, Jutta and Yvonne, Delores, CFX-1, CFX-2, and CRS-1, and Finola	GC and GC-FID	- Oil: 269 to 306 g/kg and crude protein: 238 to 280 g/kg; - Dominant FAs: LA (597 g/kg) and ALA (170 g/kg); - For all cultivars, γ-tocopherol than δ-tocopherol (2481 vs. 774 μg/g); - Anka richest in phenols (5.16 g/100 g) and CRS-1 lowest (1.37 g/100 g).	[39]
FAs, phenolics, amino acids, and cannabinoids	Untargeted metabolomics	Two accessions cultivated in Madhya Pradesh and in Himachal Pradesh	GC-MS and HPLC	- 236 metabolites detected, and 43 significantly different (*p* ≤ 0.05); - High-altitude accession: exclusive LA and ALA, higher phenols and flavonoids.	[17]
Lipids, lignans, flavanonoids, EAAs, saccha-rides, vitamins, and cannabinoids	Targeted metabolomics	Seven Chinese hemp varieties	UHPLC-QQQ-MS/MS	- 1001 metabolites. Including 43 terpenoids, 6 tannins, 149 phenolic acids, 159 lipids, 18 lignans and coumarins, 13 flavanones, 5 flavanonols, 9 anthocyanidins, 51 flavones, 55 flavonols, 103 amino acid and derivatives, 47 saccharides, 13 vitamins, and 10 cannabinoids.	[12]
FAs phenolics, crude proteins, and fibers	Targeted metabolomics	Carmaenecta, Enectaliana, and Enectarol	GC-FID and HPLC-MS	- Enectarol: highest total lipid content and best antioxidant activity. Carmaenecta: best FA profile. Enectaliana: highest crude protein and dietary fiber; - High EFAs and oleic acid. Insoluble fibers: cellulose (46%), lignin (31%), and hemicellulose (22%); - Crude proteins: ~19 g/100 g to 39 g/100 g.	[154]
FAs (LA, ALA, and γ-linolenic acid)	Metabolomics	Felina 32, Chamaeleon, Uso31, and Finola	GC-FID	- Felina 32, Chamaeleon oils: highest LA (59%) and ALA (18%); - Finola, Uso31 oils: highest γ-linolenic acid (5–6%); - All: high oxidation stability.	[52]
FAs (LA, ALA cholesterol, and tocopherol	Metabolomics	_	GC-FID	Diet including hemp seeds: - increased PUFA, including LA and ALA; - Cholesterol unaffected, except for 10% hemp seed group; - 30% hemp seed group: highest tocopherol content.	[89]
Carbohydrates, proteins, lipids, phytate, and lignin	Metabolomics	20 hemp cultivars and advanced breeding lines	HPLC and GC-FID	- Polysaccharides hulls: cellulose (22.0–36.7%) and xylan (5.7–17.1%), whereas whole seeds contained xyloglucan and pectin; - Cellulose and lignin are localized in hull; - Protein: 19.5–27%; - Lipid: 26.5–38% and omega-6:3 ratio: 2–5.	[155]
Polysaccharides (soluble)	Metabolomics	Futura 75	HPLC	- Hemp seeds: 33% uronic acids and 53% total sugars.	[11]
Terpenes (α-pinene, β-pinene, and linalool)	Metabolomics	_	GC–MS and GC-O	- 89 volatile compounds (E-nose) and 77 (GC-MS); - Most abundant: α-pinene, β-pinene, sabinene, α-phellandrene, linalool, and 1,3,8-p-menthatriene; - 16 odor-active compounds (GC-O).	[157]
Oils, terpenes (β-myrcene, and D-limonene)	Metabolomics	_	GC-FID and SPME	- Roasting (63 °C, 15 min): 45% higher extraction yield, and 80% higher oxidative stability. Oil yield: 23.09%, TPC: 121.21 mg GAE/kg, and OSI: 21.37 h; - Main volatiles: β-myrcene (3170–1178 ng/g) and D-limonene (1347–470 ng/g).	[121]
FAs, Lignanamides, and HCAAs	Untargeted metabolomics	Kongo Hanf, Spanish accession, French accession, and Italian Eletta Campana	LC-MS	- Main bioactives: cinnamic acid amides and lignanamides. Spanish accession Kongo Hanf, and French accession: highest concentrations; - Italian cultivar Eletta Campana: highest FAs.	[156]
Phenylpropanoids, HCAAs, lignanamides, and cannabinoids	Metabolomics	_	HPLC-DAD/ESI-MS/MS	- The phenolic profile was investigated and the extraction process optimized; - The total antioxidant capacity (TAC) test using an acetone–water solvent is the optimal combination to extract more phenols and have potent antioxidant activity (~259 mg TE per g extract).	[106]
Lignanamides and HCAAs	Metabolomics	CRS1 variety	HPLC-ESI-Q-TOF-MS/MS. HPLC-DAD	- 26 phenylpropionamides, including hydroxycinnamic acid amides, and lignanamides; - 25–78% increase in total phenylpropionamide in hemp seed hull after extrusion.	[110]
Polyphenols, Flavonoids, crude lipids, and fiber	Metabolomics	_	HPLC-Q-TOF-MS/MS	- Polyphenols and flavonoids explain antioxidant activity (~2720 μM Trolox equivalents); - Bioactives stable after in vitro digestion.	[113]
Lipids, flavonoids, amino acids, lignans coumarins, and terpenoids	Targeted metabolomics	Hemp seeds of seven villages in Bama County	UPLC-MS/MS	- 1020 metabolites, including 177 flavonoids, 156 phenolic acids, 134 lipids, 113 amino acids and derivatives, 106 alkaloids, 85 organic acids, 65 nucleotides and derivatives, 39 lignans and coumarins, 12 terpenoids, and 7 tannins; - DPPH free radical activity: 77.19– 91.97%.	[50]
Phytosterol (β-sitosterol, and campesterol)	Metabolomics	Italian (IT) and Extra-European (EE) varieties	HRGC and HPLC-GC-FID	- Most abundant: β-sitosterol and campesterol; - β-sitosterol: 64.5–68% of total phytosterols.	[83]
FAs (ALA, LA), polyphenols, proteins, polysaccarides, and bioactive compounds	Metabolomics	Santhica 27, Fedora 32, Felina 32, Futura 75, Tygra, Bialobrzeskie, and Finola	HPLC-DAD	- Finola: highest oil, protein; - Compositions (g/100 g): oil: 8.5–29%, protein: 12–25.4%, and carbs: 41–74.5%; - PUFAs: 53.4% LA, 12% ALA, and 3% γ-linolenic acids. Lutein: 1.5 (Futura) to 3.4 mg 100/g (Santhica).	[53]
Oils, carotenoids, phenols, and tocopherols	Metabolomics	Thirteen different commercial hemp varieties	UHPLC, HPLC-UV, and HPLC	- Oils omega-6/omega-3 ratio: 1.7–2.3. Carotenoids: 0.3–1.7 µg/g; - Phenols: 22–161 mg GAE/g. Tocopherols: 3.5–13.25 mg/100 g.	[85]
Phytocannabinoids	Metabolomics	_	UHPLC-HRGC/MS	- THC, cannabidiol, 30 cannabinoids identified. Each contributes to bioactivity and chemical profile.	[158]
PL, ALA, and LA	Lipidomics	*C. sativa*	Shotgun and RPLC associated to MRM-NPLC	- Shotgun: identified PL species. RPLC: minor species. NPLC: lysophospholipids, and cardiolipins; - Camelina cake higher ALA than hemp.	[161]
FA, PL, and minor lipid classes	Lipidomics	Nine hemp varieties	HRMS	- 184 lipids: 26 FAs, 158 PLs, including rare subclasses like N-acylphosphatidylethanolamine.	[162]
DAGs and TAGs	Lipidomics	*C. sativa*	LC-Q-TOF-MS	- Camelina, flax, and hemp oils: 40 DAGs and 118 TAGs (camelina); 39 DAGs and 110 TAGs (flax); and 36 DAGs and 105 TAGs (hemp). - 27 markers for authenticity.	[167]
LPC, LPE, PC, DG, and TG	Lipidomics	*C. sativa*	HRMS	- 65 lipids in 30 commercial oil seed samples were identified.	[166]
Proteins (11S edestin, 2S al-bumin, and 7S vicilin-like)	Proteomics	Futura 75 and Finola	SDS-PAGE, 2D-gel electrophoresis, and MS	- Protein yield per growth area and variety: Crodo—53.6 ± 5.3 mg/mL (Futura 75), 56.2 ± 5.3 mg/mL (Finola); Viganella—61.2 ± 3.5 mg/mL (Futura 75), 34.9 ± 7.2 mg/mL (Finola).	[42]
Proteins (11S edestin, 2S al-bumin, 7S vicilin-like, etc.)	Proteomics	Futura	SDS-PAGE separation and nLC-ESI-MS/MS identification	- 181 expressed proteins.	[41]
Proteins (11S edestin, 2S al-bumin, 7S vicilin-like, etc.)	Proteomics	Cheungsam	2D-gel electrophoresis and MS	- 168 unique protein spots identified, categorized by biological function, such as involvement in plant metabolism and stress response.	[66]
Minerals (Fe, Zn, Mn, Cu, Mo, Ni, and Co), FAs, phytosterols, and phenols	Metabolomics and ionomics	Hemp genotype Fedora	GC-FID, ICP-OES, and RP-HPLC-DAD	- Hemp seeds, flour, and oil: 767 ± 41, 744 ± 29, and 21 ± 5 mg GAE/kg total polyphenols, resp.; - Trace elements concentration: Fe > Cu > Ni > Mn.	[40]
Minerals (P, K, Mg, Zn, Ca, Mn, and Cu) and antinutrients (phytates)	Metabolomics and ionomics	Bialobrzeskie, Carmagnola, Fedora 17, Felina 32, KC Dora, Kompolti, Santhica 27, and Tiborszallasi	UV/visible spectroscopy	- P higher in hulled seeds (1.1 g/100 g). K, Mg, Zn, Ca, Mn, and Cu higher in whole seeds; - Phytates in hulled seeds may compromise P, Fe, and Zn absorption.	[171]
Minerals (Ca, Na, K, Fe, Mn, P, and Mg), crude proteins, and FAs	Metabolomics and ionomics	Industrial hemps	GC-FID and ICP-OES	- Fe (46.7%), Mn (169.1%), Cu (29.0%), Zn (28.2%), P (41.0%), and Mg (33.7%); - Crude protein: 32.7% to 35.9%; oil: 24.3% to 28.1%.	[173]
Lipids (ALA and LA)	Metabolomics and transcriptomics	Bama county hemp seeds varieties	MS and qRT-PCR	- Key genes (e.g., FAD2) upregulated during seed development stages.	[76]
Proteins (11S edestin, 2S al-bumin, and 7S vicilin-like)	Genomics and transcriptomics	Futura	GWAS and qRT-PCR	- 11S edestins rich in arginine and glutamic acid; - Type3 edestin abundant in cysteine and methionine; - Identified 6 edestin genes, 2 2S albumin genes, and 1 7S-vicilin like gene.	[64]
Proteins (11S edestin)	Genomics, transcriptomics, and proteomics	Carmagnola	qRT-PCR and SDS PAGE	- Identified 4 edestin type1 *CsEde1A*, *CsEde1B*, *CsEde1C*, and *CsEde1D*) and 3 edestin type2 (*CsEde2A*, *CsEde2B*, and *CsEde2C*).	[67]
Health-promoting metabolites (cannflavins (A, B, and C), trigonelline, citric acid, vitexin, choline alfoscerate, and choline)	Targeted metabolomics and transcriptomics	Bama hemp seeds varieties	UHPLC–ESI–MS/MS and RNA-Seq	- Gradual decrease in gene expression and metabolite accumulation during seed development.	[151]

**Table 4:** Relevant omics studies in hemp seed by-products, grouped according to the type of by-product.

Nutrients and Bioactive Compounds	Omics Approach	Variety of Hemp	Main Methods	Main Results	Properties of Hemp Seed by-Products	Reference
11S edestin, 2S albumin, and 7S vicilin-like In whole, dehulled seed, and hull	Metabolomics and proteomics	Santhica 27 and Uso-31	LC-MS/MS and SDS-PAGE	- Average storage proteins content: 65%, 71%, and 57% for whole seeds, dehulled seeds, and hulls, resp.; - Proteins defense and stress responses abundant in hull seeds.	- The hulls can be an essential source of proteins to be used in medical or biotechnological areas.	[46]
Phenolics, oils, and proteins In whole and dehulled seed, and hull	Metabolomics	Bama and Yunma	LH-20 gel chromatography and HPLC methods	- Oil yield of seed hull: 10.2% and 9.8%, in Bama and Yunma resp.; - Oil content: 1 g/100 g after the defatting process. Protein content: 65.06% and 67.06% in Bama and Yunma resp.; - Dominant phenolics content in hull than in kernel.	- Hemp seed hull is a source of natural antioxidants; - The defatted hemp seed kernel is a source of protein.	[16]
HPI In flour	Proteomics	Carmagnola	LC-ESI-MS/MS, and SDS PAGE	- HPI content: ~86% protein, mainly edestins. Precursors of bioactive peptides; - Allergens: thaumatin-like protein and LTP eliminated by the HPI production process.	- The bioactivity of HPI hydrolysates; - The use of HPI as an ingredient for hypoallergenic foods; - Hemp flour and HPI showed a high degree of digestibility.	[176]
FAs, polyphenols, and amino acids In flour	Metabolomics	_	GC-MS, HPLC-FLD, HPLC-FLD, and GC-FID	- TPC: ~0.73–1.73 mg GAE/g; - Antiradical activity: ~1.17–3.18 mmol TEAC/100; - omega3 content: 8.1–10%.	- Durum wheat enriched with 10% hemp flour maintains excellent rheological characteristics; - Higher ω3 content and antioxidant properties.	[44]
HCAAs, caffeic acid, cannaflin C, FAs, EAAs, gliadin, and glutenin-free proteins In flour	Metabolomics	*Cannabis sativa*	UHPLC- ESI/QTOF-MS	- Protocatechuic acid, caffeic acid, hydroxycinnamic acid, and cannaflavin C. EAAs content: ~16 g/100g), dominat gliadin and glutenin-free proteins; - FAs (~8%): LA (~54%) and ALA (~16%).	- Hemp seed flour can be included in the preparation of functional foods; - It is appropriate for celiac disease foods.	[22]
HPI In meal	Proteomics	_	SDS-PAGE	- Protein recovery extraction: 60–78% (alkaline solution), 20–48%, and 21% (NaCl solution and reference, resp.); - Dominant EAAs in water solution.	- Hemp seed meal is a potential alternative source for food proteins.	[178]
HPI In meal	Proteomics	-	SDS-PAGE	- Protein recovery (~62%) with HPP and protein purity (~75%) with HPP (200 MPa) and UAE; - UAE improves the extraction of all amino acids, independently from HPP pre-treatments.	- The optimization of oil extraction methods allows the improvement of HPI biological properties; - Antimicrobial, anti-obesity, and antioxidant properties.	[45]
HPI In meal	Metabolomics and proteomics	Different industrial hempseeds cultivars from crop year 2017	SDS-PAGE, SEC-HPLC-UV/RI/MALS, CD spectroscopy and DSC, and HS-SPME-GC–MS	- HPI yield: ~24% and ~17%, from dehulled and non-dehulled hemp seed, resp.	- HPIs showed high amounts of glutamic acid, arginine, and aspartic acid; - This amino acid profile is characterized by activities property for human health; - Antioxidant and anti-inflammatory activities.	[55]
HPC In meal	Proteomics	Futura 75	SDS PAGE	- The ball milling improves the solubility of proteins extracted at pH 8.	- Hemp protein functionality, such as surface activity and solubility, can be optimized.	[183]
HPI In meal	Metabolomics	_	HPLC	- Higher ALB solubility and foaming capacity than GLB; - Emulsion forming ability similar for both proteins.	- ALB fraction could be a good ingredient for food foam formulation; - GLB and ALB in the preparation of food emulsions.	[181]
Phenols, tocopherols, FAs, proteins, and crude fibers In meal	Metabolomics	USO 31 and Futura 75	LC-DAD-ESI-MS	- TPC: 56.93–77.89 µg/100 FW), tocopherols: 81.69–101.45 mg/100 g FW), and omega-6/omega-3 ratio (3:1) in USO 31 (about two times higher than Futura 75); - Proteins: 29.88–31.44 g/100 g FW, and crude fibers: 18.39–19.67 g/100 g.	- Hemp seed meal potential re-use in the nutraceutical field; - It is rich in EAAs; - Antioxidant properties.	[23]
Hemp seed oil extract (HOE)	Metabolomics, proteomics, and transcriptomics	_	UPLC-MS, GC-MS, and RNA-Seq	- HOE effects: alterations in purine metabolism pathways, down-regulates c-MYC, and inhibition of the expression of cell cycle-related proteins.	- HOE shows functional properties inhibiting colorectal cancer cell proliferation.	[47]
Minerals (K, Mg, P, Mn, and Fe), proteins, fibers, FAs, and bioactive compounds (p-coumaric acid and syringaresinol) In oil, dehulled seeds, cake, and flour	Metabolomics and proteomics	_	ICP-MS	- Protein-46-product: amounts of K ~1431 mg/100 g; - Insoluble non-starch polysaccharide content in flour: ~39.80%; - LA content: from ~54% in the protein-85-product to ~69.5% in the hemp cream; - Dominance of p-coumaric acid, and syringaresinol metabolites.	- Hemp seed by-products meet the recommended daily reference nutrient intakes for Mg, P, Mn, and Fe; - They contribute to prevent diabetes, cardiovascular disease, cancer, inflammatory, and autoimmune diseases; - Syringaresinol is known for its antioxidant properties; - p-coumaric acid exhibits antioxidant and antimicrobial properties.	[174]

## Abbreviations

The following abbreviations are used in this manuscript:

CBD	Cannabidiol
DAD	Diode array detection
EFAs	Essential fatty acids
ESI	Electro-spray ionization
GC	Gas chromatography
GC-FID	Gas chromatography–flame ionization detection
GC-MS/O	Gas chromatography–mass spectrometry–olfactometry
GWAS	Genome-wide association study
HPC	Hemp protein concentrates
HPI	Hemp protein isolates
HPLC	High-performance liquid chromatography
HPP	High-pressure processing
HRGC	High-resolution gas chromatography
ICP-OES	Inductively coupled plasma coupled to optical emission spectrometry
MALDI-TOF	Matrix-assisted laser desorption ionization time-of-flight
MALS	Multi-angle light scattering
nLC-ESI-MS/MS	Nanoflow liquid chromatography electrospray-ionization tandem mass spectrometry
NMR	Nuclear magnetic resonance
PUFAs	Polyunsaturated fatty acids
qRT-PCR	Quantitative reverse transcription polymerase chain reaction
Q-TOF-MS	Quadrupole time-of-flight-mass spectrometry
RI	Refractive index
RNA-Seq	RNA sequencing
SEC	Size exclusion chromatography
SDS-PAGE	Sodium dodecyl sulphate-polyacrylamide gel electrophoresis
SPME	Solid-phase microextraction
THC	Δ9-tetrahydrocannabinol
UAE	Ultrasound-assisted extraction
UHPLC-QQQ-MS/MS	Ultra-high performance liquid chromatography coupled with triple quadrupole mass spectrometry
UV	Ultraviolet

## References

[1] Andre C.M., Hausman J., Guerriero G. (2016). *Cannabis sativa*: The plant of the thousand and one molecules. Front. Plant Sci..

[2] Clarke R.C., Merlin M.D. (2016). Cannabis domestication, breeding history, present day genetic diversity, and future prospects. Crit.Rev. Plant Sci..

[3] Morales P., Hurst D.P., Reggio P.H. (2017). Molecular targets of the phytocannabinoids: A complex picture. Prog. Chem. Org. Nat. Prod..

[4] Kovalchuk I., Pellino M., Rigault P., van Velzen R., Ebersbach J., Ashnest J.R., Mau M., Schranz M., Alcorn J., Laprairie R. (2020). The genomics of cannabis and its close relatives. Annu. Rev. Plant Biol..

[5] Schluttenhofer C., Yuan L. (2017). Challenges towards revitalizing hemp: A multifaceted crop. Trends Plant Sci..

[6] Callaway J.C. (2004). Hempseed as a nutritional resource: An overview. Euphytica.

[7] Tănase Apetroaei V., Pricop E.M., Istrati D.I., Vizireanu C. (2024). Hemp Seeds (*Cannabis sativa* L.) as a valuable source of natural ingredients for functional foods—A review. Molecules.

[8] House J.D., Neufeld J., Leson G. (2010). Evaluating the quality of protein from hemp seed (*Cannabis sativa L.)* products through the use of the protein digestibility-corrected amino acid score method. J. Agric. Food Chem..

[9] Sun X., Sun Y., Li Y., Wu Q., Wang L. (2021). Identification and characterization of the seed storage proteins and related genes of *Cannabis sativa* L.. Front Nutr..

[10] Farinon B., Molinari R., Costantini L., Merendino N. (2020). The seed of industrial hemp (*Cannabis sativa* L.): Nutritional quality and potential functionality for human health and nutrition. Nutrients.

[11] Julakanti S., Raja Charles A.P., Syed R., Bullock F., Wu Y. (2023). Hempseed polysaccharide (*Cannabis sativa* L.): Physicochemical characterization and comparison with flaxseed polysaccharide. Food Hydrocoll..

[12] Ning K., Hou C., Wei X., Zhou Y., Zhang S., Chen Y., Yu H., Dong L., Chen S. (2022). Metabolomics analysis revealed the characteristicm of hemp seeds varieties and metabolites responsible for antioxidant properties. Front. Plant Sci..

[13] Crini G., Lichtfouse E., Chanet G., Morin-Crini N. (2020). Applications of hemp in textiles, paper industry, insulation and building materials, horticulture, animal nutrition, food and beverages, nutraceuticals, cosmetics and hygiene, medicine, agrochemistry, energy production and environment: A review. Environ. Chem. Lett..

[14] Montero L., Fernando A. (2023). Hemp seeds: Nutritional value, associated bioactivities and the potential food applications in the Colombian context. Front. Nutr..

[15] Simopoulos A.P. (2008). The importance of the omega-6/omega-3 fatty acid ratio in cardiovascular disease and other chronic diseases. Exp. Biol. Med..

[16] Chen T., He J., Zhang J., Li X., Zhang H., Hao J., Li L. (2012). The isolation and identification of two compounds with predominant radical scavenging activity in hempseed (seed of *Cannabis sativa* L.). Food Chem..

[17] Rashid A., Ali V., Khajuria M., Faiz S., Gairola S., Vyas D. (2021). GC–MS based metabolomic approach to understand nutraceutical potential of Cannabis seeds from two different environments. Food Chem..

[18] Abrahamsen F., Reddy G., Abebe W., Gurung N. (2021). Effect of varying levels of hempseed meal supplementation on humoral and cell-mediated immune responses of goats. Animals.

[19] Majewski M., Jurgoński A. (2021). The effect of hemp (*Cannabis sativa* L.) seeds and hemp seed oil on vascular dysfunction in obese male zucker rats. Nutrients.

[20] Gulcin İ., Ozden E.M., Mutlu M., Mirzaee Z., Bingol Z., Köksal E., Alwasel S., Goren A.C. (2024). Exploring of biological activity and diverse metabolites in hemp (*Cannabis sativa*) seed oil by GC/MS, GC–FID, and LC–HRMS chromatographies. Futur. J. Pharm. Sci..

[21] Rupasinghe H.P.V., Davis A., Kumar S.K., Murray B., Zheljazkov V.D. (2020). Industrial hemp (*Cannabis sativa* subsp. *sativa*) as an emerging source for value-added functional food ingredients and nutraceuticals. Molecules.

[22] Buzzanca C., Di Stefano V., Wildman R.E.C. (2023). Hemp flour, from waste to nutritional and nutraceuticals reuse. Nutraceutical and Functional Foods.

[23] Occhiuto C., Aliberto G., Ingegneri M., Trombetta D., Circosta C., Smeriglio A. (2022). Comparative evaluation of the nutrients, phytochemicals, and antioxidant activity of two hempseed oils and their byproducts after cold pressing. Molecules.

[24] Burton R.A., Andres M., Cole M., Cowely J.M., Augustin M.A. (2022). Industrial hemp seed: From the field to value-added food ingredients. J. Cannabis Res..

[25] Gumus Z.P., Ustun Argon Z., Celenk V.U., Ertas H., Ramadan Hassanien M.F. (2022). Bioactive Phytochemicals from Hemp (*Cannabis sativa*) Seed Oil Processing By-products. Bioactive Phytochemicals from Vegetable Oil and Oilseed Processing By-products. Reference Series in Phytochemistry.

[26] Kumar S., Kushwaha R., Verma M.L., Verma M.L., Chandel A.K. (2020). Recovery and utilization of bioactives from food processing waste. Biotechnological Production of Bioactive Compounds.

[27] Nevara G.A., Giwa Ibrahim S., Syed Muhammad S.K., Zawawi N., Mustapha N.A., Karim R. (2022). Oilseed meals into foods: An approach for the valorization of oilseed by-products. Crit. Rev. Food Sci. Nutr..

[28] Sirangelo T.M., Ludlow R.A., Chenet T., Pasti L., Spadafora N.D. (2023). Multi-Omics and Genome Editing Studies on Plant Cell Walls to Improve Biomass Quality. Agriculture.

[29] Otles S., Despoudi S., Bucatariu C., Kartal C., Galanakis C.M. (2015). Food waste management, valorization, and sustainability in the food industry. Food Waste Recovery.

[30] Borrello M., Caracciolo F., Lombardi A., Pascucci S., Cembalo L. (2017). Consumers’ perspective on circular economy strategy for reducing food waste. Sustainability.

[31] Esposito B., Sessa M.R., Sica D., Malandrino O. (2020). Towards Circular economy in the agri-food sector. A systematic literature review. Sustainability.

[32] Ancuța P., Sonia A. (2020). Oil press-cakes and meals valorization through circular economy approaches: A review. Appl. Sci..

[33] Aliferis K.A., Bernard-Perron D. (2020). Cannabinomics: Application of metabolomics in Cannabis (*Cannabis sativa* L.) research and development. Front. Plant Sci..

[34] Cerrato A., Citti C., Cannazza G., Capriotti A.L., Cavaliere C., Grassi G., Marini F., Montone C.M., Paris R., Piovesana S. (2021). Phytocannabinomics: Untargeted metabolomics as a tool for cannabis chemovar differentiation. Talanta.

[35] Hazekamp A., Tejkalová K., Papadimitriou S. (2016). Cannabis: From cultivar to chemovar II—A metabolomics approach to cannabis classification. Cannabis Cannabinoid Res..

[36] Sirangelo T.M., Ludlow R.A., Spadafora N.D. (2022). Multi-Omics approaches to study molecular mechanisms in *Cannabis sativa*. Plants.

[37] Braich S., Baillie R.C., Jewell L.S., Spangenberg G.C., Cogan N.O.I. (2019). Generation of a comprehensive transcriptome atlas and transcriptome dynamics in medicinal cannabis. Sci. Rep..

[38] Guerriero G., Behr M., Legay S., Mangeot-Peter L., Zorzan S., Ghoniem M., Hausman J.F. (2017). Transcriptomic profiling of hemp bast fibers at different developmental stages. Sci. Rep..

[39] Vonapartis E., Aubin M.P., Seguin P., Mustafa A.F., Charron J.B. (2015). Seed composition of ten industrial hemp cultivars approved for production in Canada. J. Food Compos. Anal..

[40] Siano F., Moccia S., Picariello G., Russo G.L., Sorrentino G., Di Stasio M., La Cara F., Volpe M.G. (2019). Comparative study of chemical, biochemical characteristic and ATR-FTIR analysis of seeds, oil and four of the edible Fedora cultivar hemp (*Cannabis sativa* L.). Molecules.

[41] Aiello G., Fasoli E., Boschin G., Lammi C., Zanoni C., Citterio A., Arnoldi A. (2016). Proteomic characterization of hempseed (*Cannabis sativa* L.). J. Proteomics.

[42] Cattaneo C., Givonetti A., Leoni V., Guerrieri N., Manfredi M., Giorgi A., Cavaletto M. (2021). Biochemical aspects of seeds from *Cannabis sativa* L. plants grown in a mountain environment. Sci. Rep..

[43] Cattaneo C., Givonetti A., Cavaletto M. (2023). Protein mass fingerprinting and antioxidant power of hemp seeds in relation to plant cultivar and environment. Plants.

[44] Sciacca F., Virzì N., Pecchioni N., Melilli M.G., Buzzanca C., Bonacci S., Di Stefano V. (2023). Functional end-use of hemp seed waste: Technological, qualitative, nutritional, and sensorial characterization of fortified bread. Sustainability.

[45] Cabral E.M., Zhu X., Garcia-Vaquero M., Pérez-Vila S., Tang J., Gómez-Mascaraque L.G., Poojary M.M., Curtin J., Tiwari B.K. (2023). Recovery of protein from industrial hemp waste (*Cannabis sativa*, L.) using high-pressure processing and ultrasound technologies. Foods.

[46] Bárta J., Roudnický P., Jarošová M., Zdráhal Z., Stupková A., Bártová V., Krejčová Z., Kyselka J., Filip V., Říha V. (2024). Proteomic profiles of whole seeds, hulls, and dehulled seeds of two industrial hemp (*Cannabis sativa* L.) cultivars. Plants.

[47] Yu H., Chen Y., Deng J., Cai G., Fu W., Shentu C., Xu Y., Liu J., Zhou Y., Luo Y. (2024). Integrated metabolomics and proteomics analyses to reveal anticancer mechanism of hemp oil extract in colorectal cancer. J. Pharm. Biomed. Anal..

[48] Xu J., Bai M., Song H., Yang L., Zhu D., Liu H. (2022). Hemp (*Cannabis sativa* subsp. sativa) Chemical composition and the application of hempseeds in food formulations. Plant Foods Hum. Nutr..

[49] Yano H., Fu W. (2023). Hemp: A sustainable plant with high industrial value in food processing. Foods.

[50] Chen Z., Hao S., He Z., Liu J., Zhao J., Chen C., Jia G., Chen H. (2023). Widely targeted metabolomics analysis reveals the major metabolites in the hemp seeds from the longevity village of Bama, China. Ind. Crops Prod..

[51] Naim-Feil E., Elkins A.C., Malmberg M.M., Ram D., Tran J., Spangenberg G.C., Rochfort S.J., Cogan N.O.I. (2023). The Cannabis Plant as a Complex System: Interrelationships between Cannabinoid Compositions, Morphological, Physiological and Phenological Traits. Plants.

[52] Porto C.D., Decorti D., Natolino A. (2015). Potential Oil Yield, Fatty acid composition, and oxidation stability of the hempseed oil from four *Cannabis sativa* L. cultivars. J. Diet. Suppl..

[53] Irakli M., Tsaliki E., Kalivas A., Kleisiaris F., Sarrou E., Cook C.M. (2019). Effect of genotype and growing year on the nutritional, phytochemical, and antioxidant properties of industrial hemp (*Cannabis sativa* L.) seeds. Antioxidants.

[54] Lančaričová A., Kuzmiaková B., Porvaz P., Havrlentová M., Nemeček P., Kraic J. (2021). Nutritional quality of hemp seeds (*Cannabis sativa* L.) in different environments. J. Cent. Eur. Agric..

[55] Shen P., Gao Z., Xu M., Ohm J.B., Rao J., Chen B. (2020). The impact of hempseed dehulling on chemical composition, structure properties and aromatic profile of hemp protein isolate. Food Hydrocoll..

[56] Mattila P., Mäkinen S., Eurola M., Jalava T., Pihlava J.M., Hellström J., Pihlanto A. (2018). Nutritional value of commercial protein-rich plant products. Plant Foods Hum. Nutr..

[57] Aluko R.E. (2017). Hemp seed (*Cannabis sativa* L.) proteins: Composition, structure, enzymatic modification, and functional or bioactive properties. Sustainable Protein Sources.

[58] Wang X.-S., Tang C.-H., Yang X.-Q., Gao W.-R. (2008). Characterization, amino acid composition and in vitro digestibility of hemp (*Cannabis sativa* L.) proteins. Food Chem..

[59] El-Sohaimy S.A., Androsova N.V., Toshev A.D., El-Enshasy H.A. (2022). Nutritional quality, chemical, and functional characteristics of hemp (*Cannabis sativa* ssp. *sativa*) protein isolate. Plants.

[60] Wu G., Bazer F.W., Davis T.A., Kim S.W., Li P., Rhoads J.M., Satterfield M.C., Smith S.B., Spencer T.E., Yin Y. (2009). Arginine metabolism and nutrition in growth, health and disease. Amino Acids.

[61] Lin Y., Pangloli P., Meng X., Dia V.P. (2020). Effect of heating on the digestibility of isolated hempseed (*Cannabis sativa* L.) protein and bioactivity of its pepsin-pancreatin digests. Food Chem..

[62] Hu J.X., Liu M., Jing X., Na D.Q. (2007). Study on toxicological safety of hemp seed proteins. Chin. J. Health Lab. Technol..

[63] Marcone M.F. (1999). Biochemical and biophysical properties of plant storage proteins: A current understanding with emphasis on 11S seed globulins. Food Res. Int..

[64] Ponzoni E., Brambilla I.M., Galasso I. (2018). Genome-wide identification and organization of seed storage protein genes of *Cannabis sativa*. Biol. Plant..

[65] Shen P., Gao Z., Fang B., Rao J., Chen B. (2021). Ferreting out the secrets of industrial hemp protein as emerging functional food ingredients. Trends Food Sci. Technol..

[66] Park S., Seo J., Lee M. (2012). Proteomic profiling of hempseed proteins from Cheungsam. Biochimica Et Biophysica Acta (BBA). Proteins Proteom..

[67] Docimo T., Caruso I., Ponzoni E., Mattana M., Galasso I. (2014). Molecular characterization of edestin gene family in *Cannabis sativa* L.. Plant Physiol. Biochem..

[68] Zając M., Guzik P., Kulawik P., Tkaczewska J., Florkiewicz A., Migdał W. (2019). The quality of pork loaves with the addition of hemp seeds, de-hulled hemp seeds, hemp protein and hemp flour. LWT.

[69] Pojić M., Hadnađev T.D., Hadnađev M., Rakita S., Brlek T. (2015). Bread supplementation with hemp seed cake: A by-product of hemp oil processing. J. Food Qual..

[70] Grasso N., Alonso-Miravalles L., O’Mahony J.A. (2020). Composition, physicochemical and sensorial properties of commercial plant-based yogurts. Foods.

[71] Norajit K., Gu B.-J., Ryu G.-H. (2011). Effects of the addition of hemp powder on the physicochemical properties and energy bar qualities of extruded rice. Food Chem..

[72] Aiello G., Lammi C., Boschin G., Zanoni C., Arnoldi A. (2017). Exploration of potentially bioactive peptides generated from the enzymatic hydrolysis of hempseed proteins. J. Agric. Food Chem..

[73] Lammi C., Aiello G., Vistoli G., Zanoni C., Arnoldi A., Sambuy Y., Ferruzza S., Ranaldi G. (2016). A multidisciplinary investigation on the bioavailability and activity of peptides from lupin protein. J. Funct. Foods.

[74] Kwaśnica A., Teleszko M., Marcinkowski D., Kmiecik D., Grygier A., Golimowski W. (2022). Analysis of changes in the amount of phytosterols after the bleaching process of hemp oils. Molecules.

[75] Sala-Vila A., Fleming J., Kris-Etherton P., Ros E. (2022). Impact of α-linolenic acid, the vegetable ω-3 fatty acid, on cardiovascular disease and cognition. Adv. Nutr..

[76] Nie J., Ma W., Ma X., Zhu D., Li X., Wang C., Xu G., Chen C., Luo D., Xie S. (2024). Integrated transcriptomic and metabolomic analysis reveal the dynamic process of Bama hemp seed development and the accumulation mechanism of α-linolenic acid and linoleic acid. Agric. Food Chem..

[77] Sergeant S., Rahbar E., Chilton F.H. (2016). Gamma-linolenic acid, dihommo-gamma linolenic, eicosanoids and inflammatory processes. Eur. J. Pharmacol..

[78] Lu H., Li L., Zou Z., Han B., Gong M. (2024). The therapeutic potential of hemp seed oil in d-galactose-induced aging rat model was determined through the combined assessment of 1H NMR metabolomics and 16S rRNA gene sequencing. Metabolites.

[79] Lattimer J.M., Haub M.D. (2010). Effects of dietary fiber and its components on metabolic health. Nutrients.

[80] Rizzo G., Storz M.A., Calapai G. (2023). The role of hemp (*Cannabis sativa* L.) as a functional food in vegetarian nutrition. Foods.

[81] Frassinetti S., Moccia E., Caltavuturo L., Gabriele M., Longo V., Bellani L., Giorgi G., Giorgetti L. (2018). Nutraceutical potential of hemp (*Cannabis sativa* L.) seeds and sprouts. Food Chem..

[82] Rezvankhah A., Emam-Djomeh Z., Safari M., Askari G., Salami M. (2019). Microwave-assisted extraction of hempseed oil: Studying and comparing of fatty acid composition, antioxidant activity, physiochemical and thermal properties with Soxhlet extraction. J. Food Sci. Tech..

[83] Blasi F., Tringaniello C., Verducci G., Cossignani L. (2022). Bioactive minor components of Italian and Extra-European hemp seed oils. LWT.

[84] Crescente G., Piccolella S., Esposito A., Scognamiglio M., Fiorentino A., Pacifico S. (2018). Chemical composition and nutraceutical properties of hempseed: An ancient food with actual functional value. Phytochem. Rev..

[85] Izzo L., Pacifico S., Piccolella S., Castaldo L., Narváez A., Grosso M., Ritieni A. (2020). Chemical analysis of minor bioactive components and cannabidiolic acid in commercial hemp seed oil. Molecules.

[86] Aiello A., Pizzolongo F., Scognamiglio G., Romano A., Masi P., Romano R. (2020). Effects of supercritical and liquid carbon dioxide extraction on hemp (*Cannabis sativa* L.) seed oil. Int. J. Food Sci. Technol..

[87] Liang J., Appukuttan Aachary A., Thiyam-Holländer U. (2015). Hemp seed oil: Minor components and oil quality. Lipid Technol..

[88] Debier C., Larondelle Y. (2005). Vitamins A and E: Metabolism, roles and transfer to offspring. Br. J. Nutr..

[89] Rbah Y., Taaifi Y., Allay A., Belhaj K., Melhaoui R., Houmy N., Moumen A.B., Azeroual E., Addi M., Mansouri F. (2024). A comprehensive exploration of the fatty acids profile, cholesterol, and tocopherols levels in liver from laying hens fed diets containing nonindustrial hemp seed. Hindawi Sci..

[90] Warren T., McAllister R., Morgan A., Rai T.S., McGilligan V., Ennis M., Page C., Kelly C., Peace A., Corfe B.M. (2021). The interdependency and co-regulation of the vitamin D and cholesterol metabolism. Cells.

[91] Rusu I.E., Marc Vlaic R.A., Mureşan C.C., Mureşan A.E., Filip M.R., Onica B.M., Csaba K.B., Alexa E., Szanto L., Muste S. (2021). Advanced characterization of hemp flour (*Cannabis sativa* L.) from Dacia Secuieni and Zenit varieties, compared to wheat flour. Plants.

[92] Blondeau N., Lipsky R.H., Bourourou M., Duncan M.W., Gorelick P.B., Marini A.M. (2015). Alpha-linolenic acid: An omega-3 fatty acid with neuroprotective properties-ready for use in the stroke clinic?. Biomed. Res. Int..

[93] Fan Y.Y., Chapkin R.S. (1998). Importance of dietary gamma-linolenic acid in human health and nutrition. J. Nutr..

[94] Das U.N. (2007). Gamma-linolenic acid therapy of human glioma—A review of in vitro, in vivo, and clinical studies. Med. Sci. Monit..

[95] Cerino P., Buonerba C., Cannazza G., D’Auria J., Ottoni E., Fulgione A., Di Stasio A., Pierri B., Gallo A. (2020). A review of hemp as food and nutritional supplement. Cannabis Cannabinoid Res..

[96] Wen Z.-S., Xue R., Du M., Tang Z., Xiang X.-W., Zheng B., Qu Y.-L. (2019). Hemp seed polysaccharides protect intestinal epithelial cells from hydrogen peroxide-induced oxidative stress. Int. J. Biol. Macromol..

[97] Zhou Y., Chen X., Chen T., Chen X. (2022). A review of the antibacterial activity and mechanisms of plant polysaccharides. Trends Food Sci. Technol..

[98] Es-Sai B., Wahnou H., Benayad S., Rabbaa S., Laaziouez Y., El Kebbaj R., Limami Y., Duval R.E. (2025). Gamma-Tocopherol: A comprehensive review of its antioxidant, anti-Inflammatory, and anticancer properties. Molecules.

[99] Xu M., Liu K., Swaroop M., Porter F.D., Sidhu R., Firnkes S., Ory D.S., Marugan J.J., Xiao J., Southall N. (2012). delta-Tocopherol reduces lipid accumulation in Niemann-Pick type C1 and Wolman cholesterol storage disorders. J. Biol. Chem..

[100] Shibata A., Nakagawa K., Tsuduki T., Miyazawa T. (2015). Alpha-tocopherol suppresses antiangiogenic effect of delta-tocotrienol in human umbilical veinendothelial cells. J. Nutr. Biochem..

[101] Mathur P., Ding Z., Saldeen T., Mehta J.L. (2015). Tocopherols in the prevention and treatment of atherosclerosis and related cardiovascular disease. Clin. Cardiol..

[102] Wu S., Chen R., Chen J., Yang N., Li K., Zhang Z., Zhang R. (2023). Study of the Anti-Inflammatory Mechanism of β-Carotene Based on Network Pharmacology. Molecules.

[103] Heaney R.P. (2004). Phosphorus nutrition and the treatment of osteoporosis. Mayo Clin. Proc..

[104] Weaver C.M. (2013). Potassium and health. Adv Nutr..

[105] Volpe S.L. (2013). Magnesium in disease prevention and overall health. Adv. Nutr..

[106] Benkirane C., Ben M.A., Fauconnier M.L., Belhaj K., Abid M., Caid H.S., Elamrani A., Mansouri F. (2022). Bioactive compounds from hemp (*Cannabis sativa* L.) seeds: Optimization of phenolic antioxidant extraction using simplex lattice mixture design and HPLC-DAD/ESI-MS2 analysis. RSC Adv..

[107] Sirangelo T.M., Ludlow R.A., Spadafora N.D. (2023). Molecular mechanisms underlying potential pathogen resistance in *Cannabis sativa*. Plants.

[108] Chen C., Pan Z. (2021). Cannabidiol and terpenes from hemp–ingredients for future foods and processing technologies. J. Future Foods.

[109] Nahler G., Jones T.M., Russo E.B. (2019). Cannabidiol and contributions of major hemp phytocompounds to the “entourage effect”; possible mechanisms. J. Altern. Complementary Integr. Med..

[110] Leonard W., Zhang P., Ying D., Xiong Y., Fang Z. (2021). Extrusion improves the phenolic profile and biological activities of hempseed (*Cannabis sativa* L.). Food Chem..

[111] Yan X., Tang J., dos Santos Passos C., Nurisso A., Simões-Pires C.A., Ji M., Lou H., Fan P. (2015). Characterization of lignanamides from hemp (*Cannabis sativa* L.) seed and their antioxidant and acetylcholinesterase inhibitory activities. J. Agric. Food Chem..

[112] Bourjot M., Zedet A., Demange B., Pudlo M., Girard-Thernier C. (2016). In vitro mammalian arginase inhibitory and antioxidant effects of amide derivatives isolated from thehempseed cakes (*Cannabis sativa*). Planta Med. Int. Open.

[113] Frazzini S., Torresani M.C., Roda G., Dell’Anno M., Ruffo G., Rossi L. (2024). Chemical and functional characterization of the main bioactive molecules contained in hulled *Cannabis sativa* L. seeds for use as functional ingredients. J. Agric. Food Res..

[114] Dias M.C., Pinto D.C.G.A., Silva A.M.S. (2021). Plant flavonoids: Chemical characteristics and biological activity. Molecules.

[115] Augustyńska-Prejsnar A., Topczewska J., Ormian M., Sokołowicz Z. (2022). Quality of poultry roast enriched with hemp seeds, hemp oil, and hemp flour. Foods.

[116] Yang Y., Lewis M.M., Bello A.M., Wasilewski E., Clarke H.A., Kotra L.P. (2017). *Cannabis sativa* (hemp) seeds, Δ9-tetrahydrocannabinol, and potential overdose. Cannabis Cannabinoid Res..

[117] Singh D., Singh Raghuvanshi R., Dutta A., Kumar A. (2022). Nutritional qualities of hemp seed (*Cannabis sativa* L.): An underutilized source of protein and fat. Pharma Innov. J..

[118] Przybylska-Balcerek A., Frankowski J., Graczyk M., Niedziela G., Sieracka D., Wacławek S., Sázavská T.H., Buśko M., Szwajkowska-Michałek L., Stuper-Szablewska K. (2024). Profile of polyphenols, fatty acids, and terpenes in Henola hemp seeds depending on the method of fertilization. Molecules.

[119] Frankowski J., Przybylska-Balcerek A., Graczyk M., Niedziela G., Sieracka D., Stuper-Szablewska K. (2023). The Effect of mineral fertilization on the content of bioactive compounds in hemp seeds and oil. Molecules.

[120] Chang Z.-Y., Chen C.-W., Tsai M.-J., Chen C.-C., Alshetaili A., Hsiao Y.-T., Fang J.-Y. (2023). The elucidation of structure–activity and structure-permeation relationships for the cutaneous delivery of phytosterols to attenuate psoriasiform inflammation. Int. Immunopharmacol..

[121] Mansouri F., Allay A., Moumen A.B., Benkirane C., Taaifi Y., Belhaj K., Addi M., Hano C., Fauconnier M.L., Caid H.S. (2023). Laboratory-scale optimization of hemp seed roasting temperature and time for producing a high-quality pressed oil. J. Food Process. Preserv..

[122] Vale T.G., Matos F.J.A., de Lima T.C.M., Viana G.S.B. (1999). Behavioral effects of essential oils from Lippia alba (Mill.) N.E. brown chemotypes. J. Ethnopharmacol..

[123] Yu L., Yan J., Sun Z. (2017). D-limonene exhibits anti-inflammatory and antioxidant properties in an ulcerative colitis rat model via regulation of iNOS, COX-2, PGE2 and ERK signaling pathways. Mol. Med. Rep..

[124] Yu L.L., Zhou K.K., Parry J. (2005). Antioxidant properties of cold-pressed black caraway, carrot, cranberry, and hemp seed oils. Food Chem..

[125] Buscemi S., Corleo D., Di Pace F., Petroni M.L., Satriano A., Marchesini G. (2018). The Effect of Lutein on Eye and Extra-Eye Health. Nutrients.

[126] Stahl W., Sies H. (2012). β-Carotene and other carotenoids in protection from sunlight. Am. J. Clin. Nutr..

[127] Murillo A.G., Hu S., Fernandez M.L. (2019). Zeaxanthin: Metabolism, properties, and antioxidant protection of eyes, heart, liver, and skin. Antioxidants.

[128] Devi V., Khanam S. (2019). Comparative study of different extraction processes for hemp (*Cannabis sativa*) seed oil considering physical, chemical and industrial-scale economic aspects. J. Clean. Prod..

[129] Mandrioli M., Tura M., Valli E., Toschi T.G. (2023). Composition of cold-pressed hemp seed oils: Key elements of quality and authenticity. La Riv. Ital. Delle Sostanze Grasse.

[130] Santos-Zea L., Villela-Castrejón J., Gutiérrez-Uribe J.A., Mérillon J.M., Ramawat K. (2018). Bound Phenolics in Foods. Reference Series in Phytochemistry.

[131] Laverty K.U., Stout J.M., Sullivan M.J., Shah H., Gill N., Holbrook L., Deikus G., Sebra R., Hughes T.R., Page J.E. (2019). A physical and genetic map of *Cannabis sativa* identifies extensive rearrangements at the THC/CBD acid synthase loci. Genome Res..

[132] Van Bakel H., Stout J., Cote A., Tallon C., Sharpe A., Hughes T., Page J. (2011). The draft genome and transcriptome of *Cannabis sativa*. Genome Biol..

[133] Grassa C.J., Weiblen G.D., Wenger J.P., Dabney C., Poplawski S.G., Motley S.T., Michael T.P., Schwartz C.J. (2021). A new Cannabis genome assembly associates elevated cannabidiol (CBD) with hemp introgressed into marijuana. New Phytol..

[134] Hurgobin B., Tamiru-Oli M., Welling M.T., Doblin M.S., Bacic A., Whelan J., Lewsey M.G. (2021). Recent advances in *Cannabis sativa* genomics research. New Phytol..

[135] Gao S., Wang B., Xie S., Xu X., Zhang J., Pei L., Yu Y., Yang W., Zhang Y. (2020). A high quality reference genome of wild *Cannabis sativa*. Hortic. Res..

[136] Wei H., Yang Z., Niyitanga S., Tao A., Xu J., Fang P., Lin L., Zhang L., Qi J., Ming R. (2024). The reference genome of seed hemp (*Cannabis sativa*) provides new insights into fatty acid and vitamin E synthesis. Plant Commun..

[137] Woods P., Campbell B.J., Nicodemus T.J., Cahoon E.B., Mullen J.L., McKay J.K. (2021). Quantitative trait loci controlling agronomic and biochemical traits in *Cannabis sativa*. Genetics.

[138] Petit J., Salentijn E.M.J., Paulo M.-J., Denneboom C., Trindade L.M. (2020). Genetic architecture of flowering time and sex determination in hemp (*Cannabis sativa* L.): A genome-wide association study. Front. Plant Sci..

[139] Yuan Y., Wang X., Wang L., Xing H., Wang Q., Saeed M., Tao J., Feng W., Zhang G., Song X.-L. (2018). Genome-wide association study identifies candidate genes related to seed oil composition and protein content in *Gossypium Hirsutum* L.. Front. Plant Sci..

[140] Uhdre R., Coyne C.J., Bourland B., Piaskowski J., Zheng P., Ganjyal G.M., Zhang Z., McGee R.J., Main D., Bandillo N. (2025). Association study of crude seed protein and fat concentration in a USDA pea diversity panel. Plant Genome.

[141] Galasso I., Russo R., Mapelli S., Ponzoni E., Brambilla I.M., Battelli G., Reggiani R. (2016). Variability in seed traits in a collection of *Cannabis sativa* L. genotypes. Front. Plant Sci..

[142] Crossa J., Pérez-Rodríguez P., Cuevas J., Montesinos-López O., Jarquín D., de los Campos G., Burgueño J., González-Camacho J.M., Pérez-Elizalde S., Beyene Y. (2017). Genomic selection in plant breeding: Methods, models, and perspectives. Trends Plant Sci..

[143] Bernardo R. (2021). Upgrading a maize breeding program via two-cycle genome wide selection: Same cost, same or less time, and larger gains. Crop Sci..

[144] Woods P., Price N., Matthews P., McKay J.K. (2023). Genome-wide polymorphism and genic selection in feral and domesticated lineages of *Cannabis sativa*. G3 Genes|Genomes|Genet..

[145] Yoosefzadeh Najafabadi M., Torkamaneh D. (2025). Machine learning-enhanced multi-trait genomic prediction for optimizing cannabinoid profiles in cannabis. Plant J..

[146] Deguchi M., Shriya K., Potlakayala S., George H., Proano R., Sheri V., Curtis W.R., Rudrabhatla S. (2020). Metabolic engineering strategies of industrial hemp (*Cannabis sativa* L.): A brief review of the sdvances and challenges. Front. Plant Sci..

[147] Zhang X., Xu G., Cheng C., Lei L., Sun J., Xu Y., Deng C., Dai Z., Yang Z., Chen X. (2021). Establishment of an Agrobacterium-mediated genetic transformation and CRISPR/Cas9-mediated targeted mutagenesis in hemp (*Cannabis Sativa* L.). Plant Biotechnol. J..

[148] Shiels D., Prestwich B.D., Koo O., Kanchiswamy C.N., O’Halloran R., Badmi R. (2022). Hemp genome editing—Challenges and opportunities. Front. Genome Ed..

[149] Turner T.R., James E.K., Poole P.S. (2013). The plant microbiome. Genome Biol..

[150] Koberl M., Schmidt R., Ramadan E.M., Bauer R., Berg G. (2013). The microbiome of medicinal plants: Diversity and importance for plant growth, quality, and health. Front. Microbiol..

[151] Duan M., Rao M.J., Li Q., Zhao F., Fan H., Li B., He D., Han S., Zhang J., Wang L. (2024). Metabolomic and transcriptomic analyses provide new insights into health-promoting metabolites from Cannabis seeds growing in the Bama region of China. Agronomy.

[152] Markley J.L., Brüschweiler R., Edison A.S., Eghbalnia H.R., Powers R., Raftery D., Wishart D.S. (2017). The future of NMR-based metabolomics. Curr. Opin. Biotechnol..

[153] Siudem P., Zielińska Z., Kowalska V., Paradowska K. (2022). 1H NMR and chemometric methods in verification of hemp-seed oil quality. J. Pharm. Biomed. Anal..

[154] Rosso E., Armone R., Costale A., Meineri G., Chiofalo B. (2024). Hemp seed (*Cannabis sativa* L.) varieties: Lipids profile and antioxidant capacity for monogastric nutrition. Animals.

[155] Schultz C.J., Lim W.L., Khor S.F., Neumann K.A., Schulz J.M., Ansari O., Skewes M.A., Burton R.A. (2020). Consumer and health-related traits of seed from selected commercial and breeding lines of industrial hemp, *Cannabis sativa* L.. J. Agric. Food Res..

[156] Padilla-González G.F., Rosselli A., Sadgrove N.J., Cui M., Simmonds M.S.J. (2023). Mining the chemical diversity of the hemp seed (*Cannabis sativa* L.) metabolome: Discovery of a new molecular family widely distributed across hemp. Front. Plant Sci..

[157] Jeong H., Yoon S., Jo S.M., Hong S.J., Ban Y., Park H., Youn M.Y., Shin E.C. (2024). Chemosensory of hemp seed oil extracted with hemp seed (*Cannabis sativa* L.) roasted under various conditions using electronic sensors and GC–MS/Olfactometry. Food Chem..

[158] Citti C., Linciano P., Panseri S., Vezzalini F., Forni F., Vandelli M.A., Cannazza G. (2019). Cannabinoid profiling of hemp seed oil by liquid chromatography coupled to high-resolution mass spectrometry. Front. Plant Sci..

[159] Han X.L., Gross R.W. (1994). Electrospray-ionization mass spectroscopic analysis of human erythrocyte plasma-membrane phospholipids. Proc. Natl. Acad. Sci. USA.

[160] Han X.L., Gross R.W. (2003). Global analyses of cellular lipidomes directly from crude extracts of biological samples by ESI mass spectrometry: A bridge to lipidomics. J. Lipid Res..

[161] Buré C., Solgadi A., Yen-Nicolay S., Bardeau T., Libong D., Abreu S., Chaminade P., Subra-Paternault P., Cansell M. (2016). Electrospray mass spectrometry as a tool to characterize phospholipid composition of plant cakes. Eur. J. Lipid Sci. Technol..

[162] Cerrato A., Aita S.A., Cannazza G., Cavaliere C., Cavazzini A., Citti C., Montone C.M., Taglioni E., Laganà A. (2024). One-phase extraction coupled with photochemical reaction allows the in-depth lipid characterization of hempseeds by untargeted lipidomics. Talanta.

[163] Rustam Y.H., Reid G.E. (2018). Analytical challenges and recent advances in mass spectrometry based lipidomics. Anal Chem..

[164] Han X.L., Gross R.W. (2005). Shotgun lipidomics: Electrospray ionization mass spectrometric analysis and quantitation of cellular lipidomes directly from crude extracts of biological samples. Mass Spectrom. Rev..

[165] Cerrato A., Capriotti A.L., Montone C.M., Aita S.E., Cannazza G., Citti C., Piovesana S., Aldo L. (2021). Analytical Methodologies for Lipidomics in Hemp Plant. Mass Spectrometry-Based Lipidomics: Methods and Protocols.

[166] Bakhytkyzy I., Hewelt-Belka W., Kot-Wasik A. (2023). A comprehensive lipidomic analysis of oilseeds using LC-Q-TOF-MS and dispersive micro-solid phase (D-μ-SPE) extraction techniques. J. Food Compos. Anal..

[167] Kozub A., Nikolaichuk H., Przykaza K., Tomaszewska-Gras J., Fornal E. (2023). Lipidomic characteristics of three edible cold-pressed oils by LC/Q-TOF for simple quality and authenticity assurance. Food Chem..

[168] Lopez C., Novales B., Rabesona H., Weber M., Chardot T., Anton M. (2021). Deciphering the properties of hemp seed oil bodies for food applications: Lipid composition, microstructure, surface properties and physical stability. Food Res. Int..

[169] Küllenberg D., Taylor L.A., Schneider M., Massing U. (2012). Health effects of dietary phospholipids. Lipids Health. Dis..

[170] Salt D.E., Baxter I., Lahner B. (2008). Ionomics and the study of the plant ionome. Annu Rev Plant Biol..

[171] Esteban J.I.A., Torija-Isasa M.E., Sánchez-Mata M.C. (2022). Mineral elements and related antinutrients, in whole and hulled hemp (*Cannabis sativa* L.) seeds. J. Food Compos. Anal..

[172] Skoog D.A., Holler F.J., Crouch S.R. (2017). Principal of Instrumental Analysis.

[173] Lan Y., Zha F., Peckrul A., Hanson B., Johnson B., Rao J., Chen B. (2019). Genotype x environmental effects on yielding ability and seed chemical composition of industrial hemp (*Cannabis sativa* L) varieties grown in North Dakota, USA. J. Am. Oil Chem. Soc..

[174] Ramos-Sanchez R., Hayward N.J., Henderson D., Duncan G.J., Russell W.R., Duncan S.H., Neacsu M. (2025). Hemp seed-based foods and processing by-products are sustainable rich sources of nutrients and plant metabolites supporting dietary biodiversity, health, and nutritional needs. Foods.

[175] Righetti P.G., Fasoli E., Boschetti E. (2011). Combinatorial peptide ligand libraries: The conquest of the ‘hidden proteome’ advances at great strides. Electrophoresis.

[176] Mamone G., Picariello G., Ramondo A., Nicolai M.A., Ferranti P. (2019). Production, digestibility and allergenicity of hemp (*Cannabis sativa* L.) protein isolates. Food Res. Int..

[177] Dong X., Rawiwan P., Middleditch M., Guo G., Woo M.W., Quek S.Y. (2024). Effects of protein variations by different extraction and dehydration approaches on hempseed protein isolate: Protein pattern, amino acid profiling and label-free proteomics. Food Chem..

[178] Cabral E.M., Poojary M.M., Lund M.N., Curtin J., Fenelon M., Tiwari B.K. (2022). Effect of solvent composition on the extraction of proteins from hemp oil processing stream. J. Sci. Food Agric..

[179] Zha F., Dong S., Rao J., Chen B. (2019). The structural modification of pea protein concentrate with gum Arabic by controlled Maillard reaction enhances its functional properties and flavor attributes. Food Hydrocoll..

[180] Dupree E.J., Jayathirtha M., Yorkey H., Mihasan M., Petre B.A., Darie C.C. (2020). A critical review of bottom-up proteomics: The good, the bad, and the future of this field. Proteomes.

[181] Malomo S.A., Aluko R.E. (2015). A comparative study of the structural and functional properties of isolated hemp seed (*Cannabis sativa* L.) albumin and globulin fractions. Food Hydrocoll..

[182] Decuyper I.I., Van Gasse A.L., Cop N., Sabato V., Faber M.A., Mertens C., Bridts C.H., Hagendorens M.M., De Clerck L., Rihs H.P. (2017). *Cannabis sativa* allergy: Looking through the fog. Allergy.

[183] Julakanti S., Charles A.P.R., Zhao J., Bullock F., Syed R., Myles Y., Wu Y. (2023). Hempseed protein (*Cannabis sativa* L.): Influence of extraction pH and ball milling on physicochemical and functional properties. Food Hydrocoll..

[184] Suchintita D.R., Tiwari B.K., Chemat F., Garcia-Vaquero M. (2022). Impact of ultrasound processing on alternative protein systems: Protein extraction, nutritional effects and associated challenges. Ultrason. Sonochem..

[185] Pandita D., Pandita A., Wani S.H., Abdelmohsen S.A.M., Alyousef H.A., Abdelbacki A.M.M., Al-Yafrasi M.A., Al-Mana F.A., Elansary H.O. (2021). Crosstalk of multi-omics platforms with plants of therapeutic importance. Cells.

[186] Huo Q., Song R., Ma Z. (2024). Recent advances in exploring transcriptional regulatory landscape of crops. Front. Plant Sci..

[187] Depuydt T., De Rybel B., Vandepoele K. (2023). Charting plant gene functions in the multi-omics and single-cell era. Trends Plant Sci..

[188] Amer B., Baidoo E.E.K. (2021). Omics-Driven Biotechnology for Industrial Applications. Front. Bioeng. Biotechnol..

[189] Gong M., Lu H., Li L., Feng M., Zou Z. (2023). Integration of transcriptomics and metabonomics revealed the protective effects of hemp seed oil against methionine-choline-deficient diet-induced non-alcoholic steatohepatitis in mice. Food Funct..

